# Prioritizing surveillance activities for certification of yaws eradication based on a review and model of historical case reporting

**DOI:** 10.1371/journal.pntd.0006953

**Published:** 2018-12-04

**Authors:** Christopher Fitzpatrick, Kingsley Asiedu, Anthony W. Solomon, Oriol Mitja, Michael Marks, Patrick Van der Stuyft, Filip Meheus

**Affiliations:** 1 Department of Control of Neglected Tropical Diseases, World Health Organization, Geneva, Switzerland; 2 Barcelona Institute for Global Health, University of Barcelona, Barcelona, Spain; 3 Department of Clinical Research, London School of Hygiene & Tropical Medicine, London, United Kingdom; 4 Department of Public Health, Ghent University, Ghent, Belgium; 5 Institute of Tropical Medicine, Antwerp, Belgium; University of Warwick, UNITED KINGDOM

## Abstract

**Background:**

The World Health Organization (WHO) has targeted yaws for global eradication. Eradication requires certification that all countries are yaws-free. While only 14 Member States currently report cases to WHO, many more are known to have a history of yaws and some of them may have ongoing transmission. We reviewed the literature and developed a model of case reports to identify countries in which passive surveillance is likely to find and report cases if transmission is still occurring, with the goal of reducing the number of countries in which more costly active surveillance will be required.

**Methods:**

We reviewed published and unpublished documents to extract data on the number of yaws cases reported to WHO or appearing in other literature in any year between 1945 and 2015. We classified countries as: a) having interrupted transmission; b) being currently endemic; c) being previously endemic (current status unknown); or d) having no history of yaws. We constructed a panel dataset for the years 1945–2015 and ran a regression model to identify factors associated with some countries not reporting cases during periods when there was ongoing (and documented) transmission. For previously endemic countries whose current status is unknown, we then estimated the probability that countries would have reported cases if there had in fact been transmission in the last three years (2013–2015).

**Results:**

Yaws has been reported in 103 of the 237 countries and areas considered. 14 Member States and 1 territory (Wallis and Futuna Islands) are currently endemic. 2 countries are believed to have interrupted transmission. 86 countries and areas are previously endemic (current status unknown). Reported cases peaked in the 1950s, with 55 countries reporting at least one case in 1950 and a total of 2.35 million cases reported in 1954. Our regression model suggests that case reporting during periods of ongoing transmission is positively associated with socioeconomic development and, in the short-term, negatively associated with independence. We estimated that for 66 out of the 86 previously endemic countries whose current status is unknown, the probability of reporting cases in the absence of active surveillance is less than 50%.

**Discussion:**

Countries with a history of yaws need to be prioritized so that international resources for global yaws eradication may be deployed efficiently. Heretofore, the focus has been on mass treatment in countries currently reporting cases. It is also important to undertake surveillance in the 86 previously endemic countries for which the current status is unknown. Within this large and diverse group, we have identified a group of 20 countries with more than a 50% probability of reporting cases in the absence of active surveillance. For the other 66 countries, international support for active surveillance will likely be required.

## Author summary

Yaws is a disabling and disfiguring disease. When the World Health Organization (WHO) was established in 1948, yaws was among the major public health problems that the new health agency chose to prioritize. In 2013, it formally targeted yaws for global eradication. While only 14 Member States currently report cases to WHO, many more are known to have a history of yaws and some of them may have ongoing transmission. Eradication requires certification that all countries are free of yaws. Certification, in turn, requires surveillance–and in some settings this may require population surveys or purposive case search. We reviewed the historical literature and developed a statistical model to better understand what factors were associated with some countries not reporting cases despite (likely) ongoing transmission. There are at least 86 countries or areas that stopped reporting cases but where yaws may still be present. Our model identified socioeconomic development and independence as factors associated with case reporting. Within the large and diverse group of countries with a history of yaws, we have identified a group of 20 countries with more than a 50% probability of reporting cases in the absence of active surveillance. For the other 66 countries, international support for active surveillance will likely be required.

## Introduction

The endemic treponematoses are a group of chronic bacterial infections. This group is made up of: yaws, caused by *Treponema pallidum* subsp. *pertenue*; endemic syphilis (also known as bejel), caused by *T*. *pallidum* subsp. *endemicum*; and pinta, caused by *T*. *carateum*. Of these, yaws produces the highest burden of disease globally. It is transmitted through direct skin-to-skin contact. In its primary and secondary (early) stages it causes lesions of the skin (especially on the face and feet), cartilage and bones, resulting in pain as well as social stigma. About 10% of untreated cases suffer tertiary (late-stage) yaws, with permanent disability and disfigurement of the face, lower limbs and hands [1].

In 1948, when the World Health Organization (WHO) was established, endemic treponematoses were among the major public health problems that the new health agency chose to prioritize. The second (1949) World Health Assembly (WHA) adopted resolution 2.36 to address endemic treponematoses. The extensive geographical range of these infections and the high morbidity and disability they caused justified this urgency. In 1950, WHO estimated that 160 million people were infected with yaws [2].

WHO- and UNICEF-led initiatives of 1948–1953 targeted yaws in Bechuanaland (Botswana), Ecuador, Haiti, India, Indonesia, Lao People’s Democratic Republic, Liberia, Paraguay, Philippines and Thailand [3]. Success in those initial pilot projects supported the planning of mass treatment campaigns using injectable penicillin in 46 countries from 1953–1963. These campaigns reduced the estimated global prevalence of infection from 50 million to 2.5 million by 1964 [4].

At the time, no formal certification process to confirm local elimination had been developed. Vertical yaws programmes were progressively integrated into national primary health care systems. By 1995 WHO estimated the global prevalence at 460 000 infectious cases. Over 300 000 new cases were reported by 13 countries during 2008 to 2012 [2]. It is difficult to ascertain whether the countries that stopped reporting yaws cases did so due to the interruption of transmission or simply the interruption of reporting.

The endemic treponematoses are so-called “diseases of poverty”, and human development, including economic growth, poverty reduction, improved access to health care and education, and improvements in access to water and sanitation naturally help to eliminate them by eliminating conditions which favour ongoing transmission. Interruption of transmission of endemic syphilis in Bosnia-Herzegovina, for example, was achieved by mass treatment campaigns “against a background of rapid socioeconomic change in the affected population, along with the creation of modern health services to cover the entire population” [5]. Among the factors to which are attributed the recession of yaws in Sri Lanka are: use of soap, improved water, and extended roads [6].

In 2013, the sixty-sixth WHA adopted resolution 66.12, targeting the eradication of yaws by 2020. Eradication is the “permanent reduction to zero of the worldwide incidence of an infection caused by a specific agent as a result of deliberate efforts; intervention measures are no longer needed” [7]. A global eradication programme therefore requires certifying that all countries are free of the disease. To achieve global certification of guinea worm disease (dracunculiasis) eradication, for example, WHO has been formally certifying every individual country, even if no indigenous case has ever been recorded there [8]. However, different countries will require different levels of intensity in the surveillance activities undertaken to proceed through the stages of certification.

A country reporting zero new indigenous yaws cases over a complete calendar year is considered to be in the first, pre-certification stage. In some countries, active community-based surveillance and periodic case searches, including cash rewards, may be required to detect new cases. In others, passive surveillance through Integrated Disease Surveillance and Response or other existing systems, plus ad hoc case searches, may suffice.

It is currently recommended that a country will enter into certification only if: 1) it has reported 0 new indigenous cases for 3 or more consecutive calendar years; 2) it shows no evidence of recent transmission (no children aged 1–5 years with rapid plasma reagin sero-reactivity); and 3) it documents negative polymerase chain reaction (PCR) for *Treponema pallidum* subspecies *pertenue* in suspected lesions [9]. Certification activities include a visit by an international team to assess the adequacy of the surveillance system, review records of rumour cases and interview health workers and affected populations.

After certification, a country will automatically enter into post-certification. Some surveillance will need to be maintained until global eradication is achieved. In countries with strong health systems, this may again be passive surveillance; in others, relatively active but localised surveillance may be required, particularly if there is transmission of yaws in a neighbouring country.

The cost of global certification of guinea worm disease eradication has not been trivial. Pre-certification and certification/post-certification have cost millions of US$ a year [10]. Lessons from that programme suggest that “It is important to reduce the cost of certification and at the same time to ensure that interruption of the disease transmission has really taken place. It is also important not to overload a country’s health system with work when the disease is no longer a public health problem and interest in it has waned” [11].

In the case of yaws eradication, the group of countries that will have to undertake surveillance is larger and more diverse, and the cost could be higher. In this study, we reviewed the literature for reports of active yaws cases and developed a descriptive model to identify countries in which passive surveillance is likely to find cases if transmission is still occurring, and thereby provide evidence to inform a reduction in the number of countries in which augmented efforts and more costly active surveillance will be required.

## Methods

A literature search was conducted on 21 September 2016 for articles published between 1 January 1945 and 21 September 2016. We updated the search on 21 December 2017.

We searched for articles on yaws (frambesia). We considered other variations on the name, based on the languages of the major colonial empires of the post-World War II era: Dutch (framboesia), French (pian), Spanish (buba) and Portuguese (bouba). Given the post-colonial alignment of many African states with the Union of Soviet Socialist Republics (USSR), we confirmed that search engines were capturing transliterated results for *фрамбезия OR frambeziya*. We checked also for “pathek” (specific to Indonesia), “parangi” (specific to Sri Lanka), “gangosa” and “goundou” (referring to particular clinical manifestations).

In PubMed, our search included the following terms: yaws[MeSH] OR yaws[Title] OR treponematoses[Title] OR “Treponema pertenue”[Title] OR frambesi*[Title] OR framboesi*[Title] OR pian[Title] OR buba[Title] OR bouba[Title]. In Global Health–CABI, we applied the same search terms but using “yaws” as a subject term rather than a MeSH term.

The search terms *yaws OR treponematoses OR “Treponema pertenue” OR frambesia OR framboesia OR pian OR buba OR bouba* were applied (without limit to field) in the WHO Institutional Repository for Information Sharing (WHO IRIS), containing all the published information produced by WHO, including proceedings of the WHA and WHO Executive Board, monographs, periodicals, unpublished technical documents, press releases, fact sheets and administrative documents of the governing bodies. From the Pan American Health Organization Institutional Repository for Information Sharing (PAHO IRIS) we extracted all “Health in the Americas” reports containing any of the above search terms, as not all of these regional reports were available through WHO IRIS.

For citations from developing countries and regions and articles published in journals that are less frequently indexed in PubMed and Global Health–CABI, we performed the same search within Global Index Medicus, and the regional indexes for Africa (AIM), Latin America and the Caribbean (LILACS), South-East Asia (IMSEAR), Eastern Mediterranean (IMEMR), and Western Pacific (WPRIM).

We also searched for the same terms in the WHO Archives, containing mainly textual paper documents, such as correspondence and mission reports. For reasons of confidentiality, these records can be consulted only 20 years after their production, so this search was limited to documents dated 7 April 1948 (the date WHO was established) to 21 December 1997.

We complemented these sources with those in the Global Infectious Disease Epidemiology Network review, which extracted reported case numbers from health ministry publications and ProMED, an internet-based reporting system on infectious disease outbreaks [12].

We included documents reporting active primary/secondary cases with clinical manifestations, regardless of whether these were laboratory-confirmed or not. Active cases include both infectious yaws (i.e., presenting with skin lesions) and non-infectious yaws (i.e., presenting with cartilage and bone but no skin lesions). Reports of latent cases (i.e., infections without any lesions, detectable only by serology) or late / tertiary cases with permanent clinical manifestations (e.g., gangosa, goundou) but no active disease were also included, but classified as reports of zero new cases.

Case reports of imported cases only were not included. However, if the country of origin was reported, we checked to ensure that the country of origin was nonetheless listed in our database as a country with a history of yaws.

We reviewed all titles and available abstracts. If available abstracts were relevant but did not contain the number of reported cases or the countries in which those cases occurred, we attempted to retrieve the full text. If full texts did not contain both the number and year(s) of cases, but instead only a general statement about past endemicity, we marked the country as previously endemic but did not further consider the reference for analysis. If the full text was not available, we classified the reference as “full text not available”. If no abstract or full text was available, we classified the reference as “abstract and full text not available” and it was not further considered in the present study.

One author extracted from each document (abstract and, if available, full text) the country and year of case reports and number of cases reported and entered data into an Excel spreadsheet. Separately, the same author extracted information on the mass treatment campaigns of 1946–1963, both national and subnational, as summarized in two WHO documents [3,13]. After 1963, programs were implemented only sporadically [14].

When cases were reported for periods of multiple years, we recorded the average number of cases per year for each year (assuming that case reporting occurred in each year of the period). In the case of inconsistencies between sources reporting on the same country and year, we took the higher number, assuming that lower numbers represented partial reports (i.e., less than 12 months’ reporting or less than national coverage).

We classified all countries and areas for the year 2015, the latest year for which WHO had received case reports at the time of writing (Table 1).

**Table 1:** Grouping of countries according to yaws endemicity status in 2015.

	Subgroup	Description
A	Current status known	Countries whose endemicity status is known to WHO
*A*.*1*	Interrupted transmission	*Countries reporting 3 or more consecutive years of zero new indigenous cases*
*A*.*2*	Currently endemic	*Countries reporting less than 3 consecutive years of zero new indigenous cases*
B	Current status unknown	Countries whose endemicity status is not known to WHO
*B*.*1*	Previously endemic	*Countries with at least one indigenous case reported since 1945 but no longer reporting to WHO*
*B*.*2*	No history of case reports	*Countries with no indigenous cases reported since 1945*

We described the distribution of endemicity by WHO region and World Bank income group. Income groups are based on Gross National Income (GNI) per capita (Atlas method) of 2013 [15].

We constructed a panel dataset based on all *N* countries with a history of case reports (A.1, A.2 and B.1), over a maximum *T* = 71 years (1945–2015), as determined by the availability of data (see below). We calculated the frequency of case reporting and total number of cases reported over time. We then performed a multivariable regression of the case reporting variable on other variables with which it might be associated.

Case reporting was represented by a binary variable. It was coded as 1 in years in which a positive and specific number of cases were reported and 0 in years in which there was no positive report (0 cases or no report) or in which the specific number of cases and years were not reported. It was coded as not available (i.e. missing) in the years after the last case report for a given country.

Yaws is an endemic not an outbreak disease (transmission does not skip years). The distinction between before and after the last reported case is that before the last reported case we know that surveillance should have detected at least one case, because we know that cases must have occurred even though they were not reported—thus the variable is coded as 0 in years of no case report before the last reported case. After the last reported case, we do not know whether surveillance should have detected at least one case because we do not know if cases have occurred—thus the variable is coded as missing.

In other words, we limited ourselves to data before the last reported case, to focus on the absence/presence of case reports due to the absence/presence of adequate surveillance, rather than due to the absence/presence of new cases. By adequate surveillance, we mean surveillance that detects at least one case if cases have occurred.

We considered a generalized linear regression model with random effects. With random effects we could include time-invariant variables and make predictions beyond the countries used in the model (i.e., for those countries without case reports). The problem of omitted variable bias was not a major concern insofar as we were not trying to get estimates of the true regression coefficients, but to make predictions that were the best that the available data would allow.

In particular, we considered the following panel linear model specification:

$$\begin{array}{l} {REP}_{it}={REP}_{it-3}\beta_{1}+\log\left({gdp}_{it}\right)\beta_{2}+{IND}_{it}\beta_{3}+{IND}_{it-3}\beta_{4}+{IND}_{it-6}\beta_{5}+{IND}_{it-9}\beta_{6}\\ \;+{IND}_{it-12}\beta_{7}+{CON}_{it}\beta_{8}+{CON}_{it-3}\beta_{9}+sqrt({aids}_{it})\beta_{10}+{CAM}_{it}\beta_{11}\\ \;+{ARA}_{i}\beta_{12}+\alpha +u_{i}+\varepsilon_{t} \end{array}$$ (1)

for *i* = 1,…,*N* countries and *t* = 1,…,*T*_i_* years, where:

*REP*_*it*_ is a dummy variable indicating if there was a case report in any of the last three calendar years up to and including year *t* in country *i*, from 1947 (based on years 1945–1947) until the year of the last case report, available through this study; the choice of three years is determined by one of the criteria for yaws certification (see Introduction);

*REP*_*it-3*_ is a lagged dependent variable to model the dynamics (persistence) in case reporting, the idea being that a country is more likely to report in the current period if they reported in the previous period, in part because of recent experience with detecting the disease; a three-year lag was chosen to avoid any overlap in years between the dependent and lagged dependent variables;

*gdp*_*it*_ is a three-year moving average (mean) of expenditure-side real GDP per capita at chained PPPs, for a comparison of relative living standards across countries, available from the Penn World Tables (version 9.0) from 1950, but not for all countries [16,17]; this variable is meant to capture the overall quality and reach of surveillance systems;

*IND*_*it*_ is a dummy variable indicating whether a country gained independence from any colonial powers in any of the last three calendar years up to and including year *t*; colonial history data are available from the Correlates of War since 1945 [18]; this variable is meant to capture any political disruption to surveillance systems;

*IND*_*it-3*,_ *IND*_*it-6*,_ *IND*_*it-9*,_ and *IND*_*it-12*_ are 3-, 6-, 9- and 12-year lags of the *IND*_*it*_ variable, allowing for some persistence in political disruption to surveillance systems;

*CON*_*it*_ is a dummy variable indicating whether there was armed conflict in any of the last three calendar years up to and including year *t*, defined by at least 25 battle-related deaths; data are available from Uppsala Conflict Data Program since 1946 [19]; again, this variable is meant to capture disruptions to surveillance systems;

*CON*_*it-3*_ is a 3-year lag of the *CON*_*it*_ variable, allowing for some persistence in disruption to surveillance systems due to armed conflict;

*aids*_*it*_ is a three-year moving average (mean) of the estimated number of AIDS-related deaths per 10 000 population, available from UNAIDS since 1990, and assumed equal to 0 in the period 1945–1989 and for all countries not reporting data to UNAIDS; this variable is meant to serve as a proxy for reorientation of yaws surveillance systems toward other public health priorities in the 1990s [20];

*CAM*_*it*_ is a dummy variable indicating whether a mass campaign (national or subnational) was undertaken in any of the last three calendar years up to and including year *t*; data are based on campaigns led by WHO or UNICEF in 1948–1963 [3,13]; during years of mass campaigns, the probability of detecting and reporting cases is higher than with passive surveillance alone;

*ARA*_*i*_ is a time-invariant dummy variable for countries where Arabic is an official language; this variable is meant to correct for the fact that our literature review did not include Arabic language search terms, and that many Arabic language journals are not indexed by PubMed;

*α* is the intercept;

*u*_*i*_ is the country-specific random effect;

*ε*_*t*_ is the year-specific random effect; and

*T*_i_* is the year of the last case report for country *i*.

Logarithmic (*log*) and square root (*sqrt*) transformations were done to improve fit of the linear model by minimizing Akaike Information Criterion values.

The lagged dependent variable (*REP3*_*it-3*_) will be correlated with the error term. The regression coefficient for the lagged dependent variable (*β*_1_) will be upwardly biased and the coefficients for other variables will be downwardly biased, in absolute terms. We therefore also ran Model (2), replacing the lagged dependent variable with two new variables:

$$\begin{array}{l} {REP}_{it}=\mathrm{sqrt}({\mathrm{yrs}}_{\mathrm{it}-3})\beta_{0}+log({\mathrm{num}}_{\mathrm{it}-3})\beta_{1}+\log\left({gdp}_{it}\right)\beta_{2}+{IND}_{it}\beta_{3}+{IND}_{it-3}\beta_{4}\\ \;+{IND}_{it-6}\beta_{5}+{IND}_{it-9}\beta_{6}+{IND}_{it-12}\beta_{7}+{CON}_{it}\beta_{8}+{CON}_{it-3}\beta_{9}\\ \;+sqrt({aids}_{it})\beta_{10}+{CAM}_{it}\beta_{11}+{ARA}_{i}\beta_{12}+\alpha +u_{i}+\varepsilon_{t} \end{array}$$ (2)

where:

*yrs*_*it-3*_ is the number of years since the most recent case report (previous to *t-3*); a three-year lag was chosen to avoid any overlap in years with the dependent variable; and

*num*_*it-3*_ is the number of cases reported in the most recent case report, per 10 000 population; again, a three-year lag was chosen to avoid any overlap in years with the dependent variable.

Given that GDP data were available for a limited number of countries (and that GDP data are more likely to be missing for poorer countries), we also considered as a proxy for the quality and reach of surveillance systems, the logistic (logit) transformation of urban population share (*urb*_*it*_), with complete data since 1950 [21]:

$$\begin{array}{l} {REP}_{it}=\mathrm{sqrt}({\mathrm{yrs}}_{\mathrm{it}-3})\beta_{0}+log({\mathrm{num}}_{\mathrm{it}-3})\beta_{1}+\mathrm{logit}\left({urb}_{it}\right)\beta_{2}+{IND}_{it}\beta_{3}+{IND}_{it-3}\beta_{4}\\ \;+{IND}_{it-6}\beta_{5}+{IND}_{it-9}\beta_{6}+{IND}_{it-12}\beta_{7}+{CON}_{it}\beta_{8}+{CON}_{it-3}\beta_{9}\\ \;+sqrt({aids}_{it})\beta_{10}+{CAM}_{it}\beta_{11}+{ARA}_{i}\beta_{12}+\alpha +u_{i}+\varepsilon_{t} \end{array}$$ (3)

Using the resulting regression coefficients from Model (3), and setting the Arabic language variable to 0, we predicted the probability of case reporting in the years after the last reported case. Since the regression model was run on data from years of (presumed) ongoing transmission, the predicted probabilities for (later) years of unknown transmission should be interpreted as the probability that a given country would report cases in a given three-year period if there were in fact new cases to report.

The predicted value for the year 2015, ${\hat{REP3}}_{i2015}$, is constrained to values between 0 and 1. In the absence of mass treatment campaigns (*CAM*_*i*2015_ = 0), we interpreted ${\hat{REP3}}_{i2015}$ as the probability that a given country would “report” cases through routine, generally passive surveillance, if transmission were ongoing. We therefore used $\hat{{REP}_{i2015}}$ to identify countries in which passive surveillance is likely to be sufficient. For the sake of illustration, we set the minimum cut-off for the probability of reporting at 50%.

All data were analysed using the open-access software R [22]. The data and code are available with this paper as Supporting Information.

## Results

### Literature search

The PubMed, Global Health–CABI and Global Index Medicus searches identified 2434 items to be assessed. WHO IRIS yielded 5450 results. Given the volume of results and low yield (2 relevant documents) from the first 100 results, we limited our WHO IRIS search to the following titles: “Weekly epidemiological record” yielded 73 references; “Report on the world health situation”, 20; “Reported cases of notifiable diseases”, 12; “Country health information profiles”, 8; and “Socioeconomic and health indicators”, 12. PAHO IRIS yielded 9 reports on “Health conditions in the Americas”. WHOLIS provided 58 documents. WHO Archives yielded another 18 correspondences and mission reports.

Altogether, after removing duplicates, we identified 2392 documents. 162 of these did not have an available abstract or full-text. The remaining 2230 documents were assessed for inclusion. Full-texts were assessed for inclusion only when the year and number of case reports was not reported in the abstract. 73 full-texts were not available. Another 1744 documents were found to not contain case reports of non-imported active clinical yaws cases.

A total of 413 abstracts or full-texts had relevant data that could be extracted. The flow diagram and list of included studies, after removal of duplicates, is provided in Supporting Information S1 Fig and S2 Table.

### Status of endemicity

We describe here the status of yaws endemicity for the 194 Member States of WHO, as well as for 9 areas for which we found separate case reports in the literature: British Virgin Islands, French Guiana, Guadeloupe, Guam, Martinique, Montserrat, New Caledonia, Puerto Rico, and Wallis and Futuna Islands.

**Fig 1:**
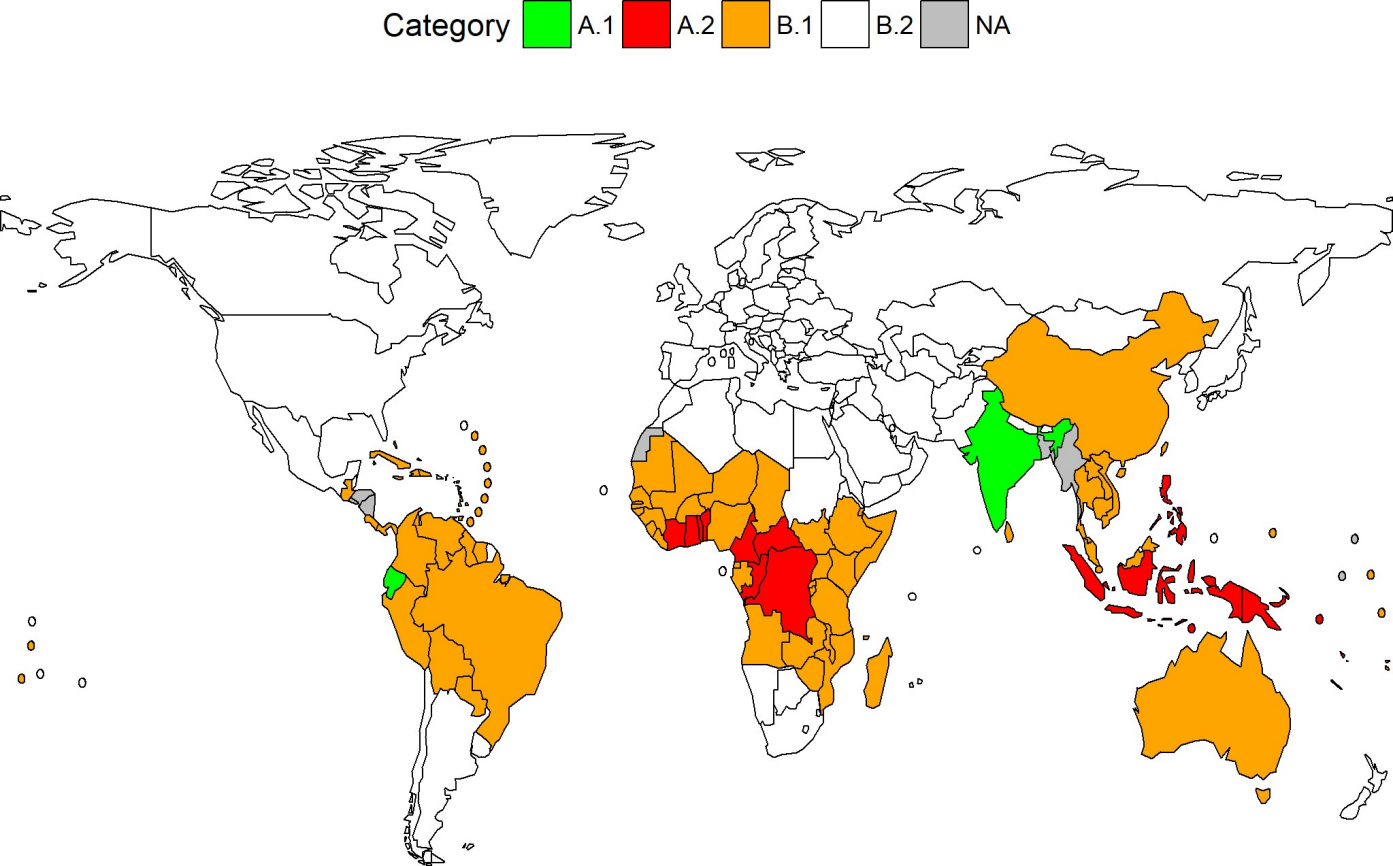
Yaws endemicity status, 2015. A.1: Interrupted transmission; A.2: Currently endemic; B.1: Previously endemic (current status unknown); B.2: No history of case reports. References to yaws were found for another 7 Member States, displayed here as not available (NA) because of non-specific case reports: Bangladesh, El Salvador, Honduras, Marshall Islands, Myanmar, Nauru and Nicaragua. In addition to Member States, there are 9 countries or areas with a history of yaws for which categorization is not displayed on this map: British Virgin Islands, French Guiana, Guadeloupe, Guam, Martinique, Montserrat, New Caledonia, Puerto Rico, and Wallis and Futuna Islands. Created using R and World Health Organization shapefiles under Creative Commons license (CC-BY).

Of the 203 countries and areas considered, 96 have reported active clinical non-imported yaws cases since 1945. Fig 1 displays the endemicity status for the 96 countries with some history of yaws case reports, as defined in this study. References to yaws were found for another 7 Member States, not displayed here because no specific case reports could be extracted from the available references. These 7 Member States are: Bangladesh (Chittagong Hills), El Salvador, Honduras, Marshall Islands, Myanmar (“Northern and Southern Burma”), Nauru and Nicaragua [12,23–26].

Two of the 96 countries (Ecuador and India) report having interrupted transmission, although one (Ecuador) has not yet been certified by WHO. 14 WHO Member States and one non-Member State (Wallis and Futuna Islands) are currently reporting yaws cases. These are all located within the African, South-East Asian and Western Pacific Regions (Table 2).

**Table 2:** Yaws endemicity status by WHO region, 2015.

Region	A: Current status known		B: Current status unknown		Total countries and areas
	A.1: Interrupted transmission	A.2: Currently endemic	B.1: Previously endemic	B.2: No history of case reports	
Africa	0	8	28	11	47
Americas	1	0	24	10	35
Eastern Mediterranean	0	0	2	19	21
Europe	0	0	0	53	53
South East Asia	1	2	2	6	11
Western Pacific	0	4	15	8	27
Non Member States	0	1^a^	8^b^	0	9
**Total**	**2**	**15**	**79**^c^	**107**	**203**

^a^ Wallis and Futuna

^b^ British Virgin Islands, French Guiana, Guadeloupe, Guam, Martinique, Montserrat, New Caledonia, Puerto Rico, and Wallis and Futuna Islands.

^c^ References to yaws were found for another 7 Member States, not included here because of non-specific case reports: Bangladesh, El Salvador, Honduras, Marshall Islands, Myanmar, Nauru and Nicaragua.

The current status of another 79 countries and areas with a history of yaws case reports remains unknown. These previously endemic countries are widely distributed: 28 of 47 Member States in Africa, 24 of 35 in the Americas, and 15 of 27 in the Western Pacific. Europe is the only WHO Region with no history of yaws since 1945.

The 14 currently endemic WHO Member States are all low- and lower-middle income countries (Table 3). Of the 79 previously endemic countries, 23 are low income and 16 are lower-middle income; at least 35 are today upper-middle income or high income.

**Table 3:** Yaws endemicity status by World Bank income group, 2015.

Income group	A: Current status known		B: Current status unknown		Total countries and areas
	A.1: Interrupted transmission	A.2: Currently endemic	B.1: Previously endemic	B.2: No history of case reports	
Low	0	4	23	7	34
Lower-middle	1	10	16	21	48
Upper-middle	1	0	24	32	57
High	0	0	11	47	58
Not categorized by the World Bank	0	1^a^	5^b^	0	6
**Total**	**2**	**15**	**79**^c^	**107**	**203**

^a^ Wallis and Futuna

^b^ British Virgin Islands, French Guiana, Guadeloupe, Guam, Martinique, Montserrat, New Caledonia, Puerto Rico, and Wallis and Futuna Islands.

^c^ References to yaws were found for another 7 Member States, not included here because of non-specific case reports: Bangladesh, El Salvador, Honduras, Marshall Islands, Myanmar, Nauru and Nicaragua.

Counting the 79 countries with specific case reports as well as the 7 countries with non-specific case reports, there are 86 previously endemic countries (current status unknown) that could, in principle, enter into pre-certification on the basis of a single report of zero cases to WHO. This large and diverse group of countries therefore requires further sub-categorization to inform the prioritization of active surveillance towards subsequent certification by WHO.

### Frequency of case reports

**Fig 2:**
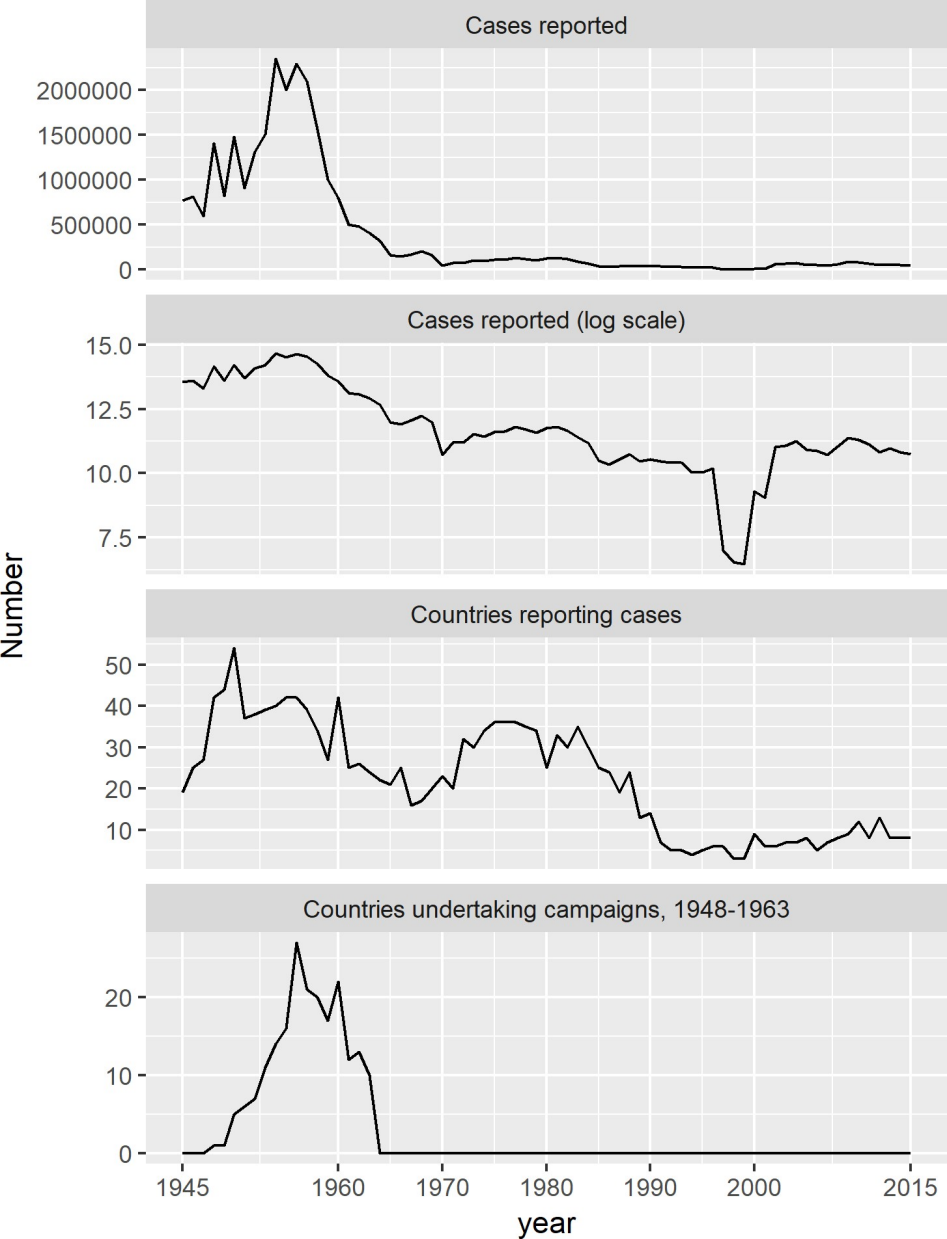
Number of yaws cases reported, and countries and areas reporting cases and undertaking mass treatment campaigns, 1945–2015. The number of countries and areas undertaking national or subnational campaigns refers to the period 1948–1963 only.

Fig 2 depicts the number of countries reporting cases and cases reported, over the years 1945–2015. 54 countries reported yaws cases in 1950. The greatest number of cases reported in a given year was 2.35 million in 1954, in the midst of mass treatment campaigns by WHO and UNICEF. When displayed against a logarithmic scale, a large but temporary drop in the number of cases reported is visible in the second half of the 1990s, when high burden countries Cote d’Ivoire and the Solomon Islands both temporarily stopped reporting cases.

**Fig 3:**
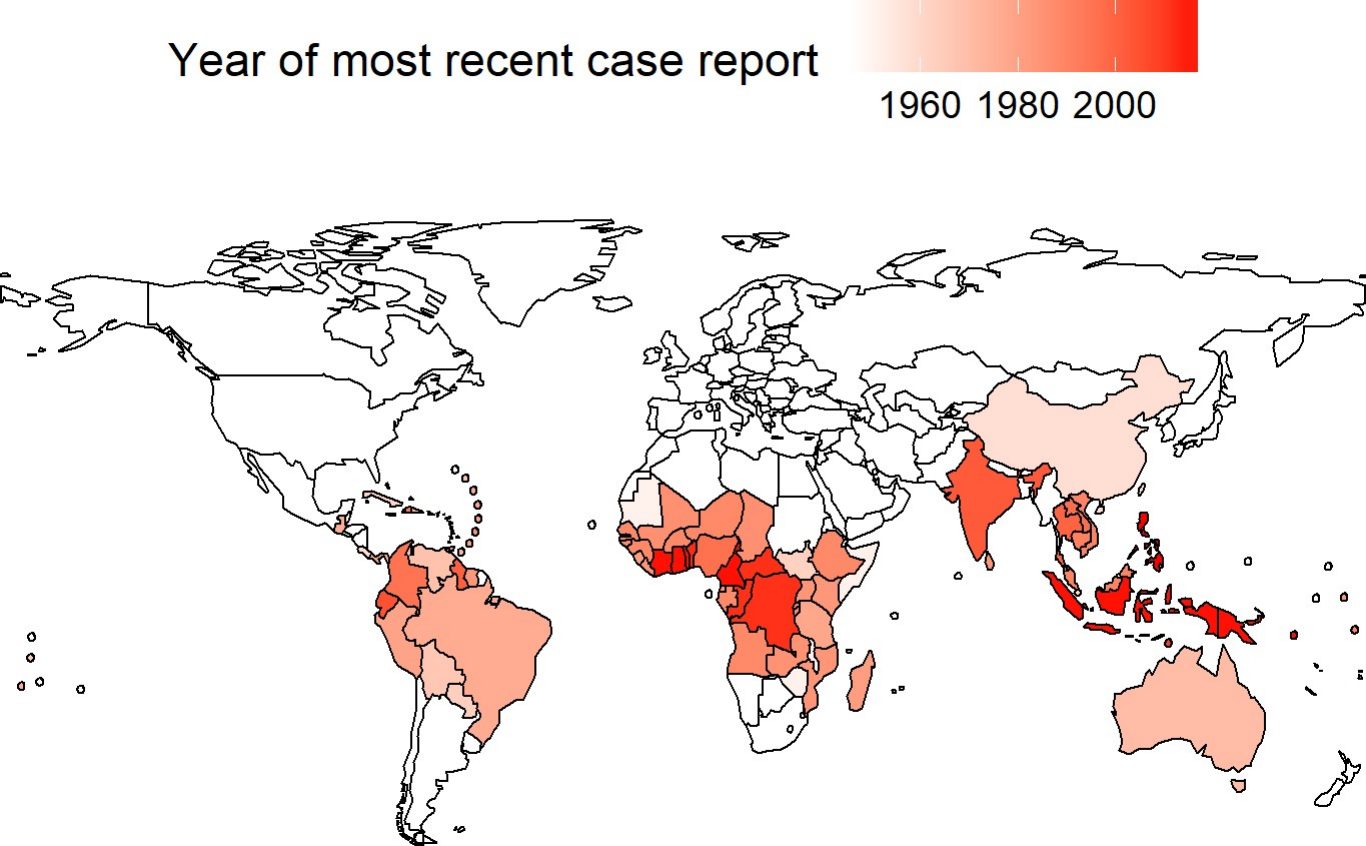
Year of most recently reported yaws case in currently or previously endemic countries/areas, 2015. In addition to Member States, there are 9 countries or areas with a history of yaws for which data are not included in this map: British Virgin Islands, Guadeloupe, French Guiana, Guam, Montserrat, Martinique, New Caledonia, Puerto Rico, and Wallis and Futuna; for Bangladesh, El Salvador, Honduras, Marshall Islands, Myanmar, Nauru, and Nicaragua we found only general references to yaws endemicity (but no case reports). Created using R and World Health Organization shapefiles under Creative Commons license (CC-BY).

Fig 3 identifies the 96 countries and areas with some history of yaws case reports (A.1, A.2 and B.1 countries) with the year of the most recently reported case. Puerto Rico last reported a case in 1945. Most countries have not reported a case since the mid-1980s. The 14 Member States considered by WHO as currently endemic have all reported at least one case since 2004. Furthermore, Wallis and Futuna Islands last reported cases in 2010. Ecuador and India last reported cases in 2005 and 2003, respectively.

The percentage of countries reporting cases before the (country-specific) most recent case report (i.e., in years of known transmission) varied from a low of 17% (3 out of 18) in 1998 to a high of 86% (12 out of 14) in 2010. 60 countries reported in fewer than 50% of years of known transmission; 24 countries, including Australia, reported in fewer than 20% of such years; 14 countries, including China (with only one report, in 1957) [27], reported in fewer than 10% of such years.

Profound change has occurred in many previously endemic countries since their most recent case reports. Most have experienced real economic growth of 100–200%. A large number of countries gained their independence from colonial powers in the 1960s. By the year 1990, 50 countries had experienced at least one year of more than 25 battle-related deaths in armed conflict. By the year 2000, 50 were reporting AIDS-related deaths of more than 1 per 10 000 population.

In the next section, we report on factors associated with case reporting in the years before the most recent case report.

### Variables associated with case reporting

The results of the regression model are presented in Table 4.

**Table 4:** Association between yaws case reporting and selected variables in the years before the most recent case report, all countries with a history of yaws. In the leftmost column, short variable descriptions are provided with the variable names as they appear in Eqs 1-3 –please refer to the Methods for a detailed description of each variable; the next three columns give the regression coefficients for each of the three model specifications corresponding to Eqs 1-3; the standard error of the estimate is reported in parentheses below the coefficient.

	Model 1	Model 2	Model 3
Intercept	-2.96***	-2.42***	1.68***
	(0.68)	(0.71)	(0.21)
Case reported in previous period, REP_t-3_	0.99***		
	(0.09)		
Average GDP per capita in current period, log(gdp_t_)	0.38***	0.46***	
	(0.09)	(0.09)	
Independence obtained in current period, IND_t_	-0.75***	-0.78***	-0.15
	(0.18)	(0.18)	(0.15)
Independence obtained in previous period, IND_t-3_	-1.36***	-1.28***	-0.49**
	(0.19)	(0.19)	(0.15)
Independence obtained in previous period, IND_t-6_	-1.41***	-1.42***	-0.70***
	(0.19)	(0.19)	(0.15)
Independence obtained in previous period, IND_t-9_	-1.21***	-1.20***	-0.66***
	(0.18)	(0.18)	(0.15)
Independence obtained in previous period, IND_t-12_	0.30	0.39*	0.59***
	(0.16)	(0.17)	(0.15)
Armed conflict in current period, CON_t_	0.33**	0.39**	0.36**
	(0.13)	(0.13)	(0.12)
Armed conflict in previous period, CON_t-3_	-0.02	0.02	0.00
	(0.13)	(0.13)	(0.13)
Average AIDS deaths in current period, per 10 000 population, sqrt(aids_t_)	-0.19***	-0.15**	-0.21***
	(0.05)	(0.05)	(0.05)
Mass treatment campaign undertaken in current period, CAM_t_	0.47**	0.29	0.04
	(0.17)	(0.17)	(0.13)
Arabic language is an official language, ARA	0.17	-0.00	0.28
	(0.63)	(0.54)	(0.47)
Number of years since most recent case report, sqrt(yrs_t-3_)		-0.36***	-0.31***
		(0.03)	(0.03)
Number of cases reported in most recent case report, per 10 000 population, log(num_t-3_)		0.07***	0.08**
		(0.02)	(0.03)
Average urban population share in current period, logit(urb_t_)			0.64***
			(0.11)
Log-Likelihood	-835.67	-788.15	-1091.27
Num. obs.	1773	1681	2232

Note

*p<0.1

**p<0.05

***p<0.01

Model (1) is applied to an unbalanced panel of 63 out of 96 countries, with a total of 1773 observations. Model (2) replaces the lagged dependent variable from Model (1). Persistence in case reporting is captured instead by the number of years since the most recent case report, and by the number of cases reported in that year.

Replacing GDP per capita with the urban population share, Model (3) is applied to an unbalanced panel of 77 out of 96 countries, with a total of 2232 observations. 19 of 96 countries and areas with some history of yaws last reported cases before 1960 and did not have sufficient observations for the specified lagged variables.

In all three models, most of the regression coefficients have the expected signs. Negatively associated with yaws case reporting are: independence (becoming less negatively associated the greater the number of years since independence), AIDS deaths per 10 000 population, and number of years since the most recent case report. Positively associated with yaws case reporting are: case reporting in the previous period or the number of cases reported in the most recent case report, mass treatment campaigns and GDP per capita or the urban population share.

It is worth noting that the coefficients on the armed conflict variable are not of the expected sign, being positively associated with case reporting. This unexpected result could be due to imprecise estimation, with armed conflict following independence in many countries. In any case, as these are meant to be predictive not explanatory models, we do not go into any detail here on the statistical significance of any individual coefficient.

### Predicted probability of case reporting

**Fig 4:**
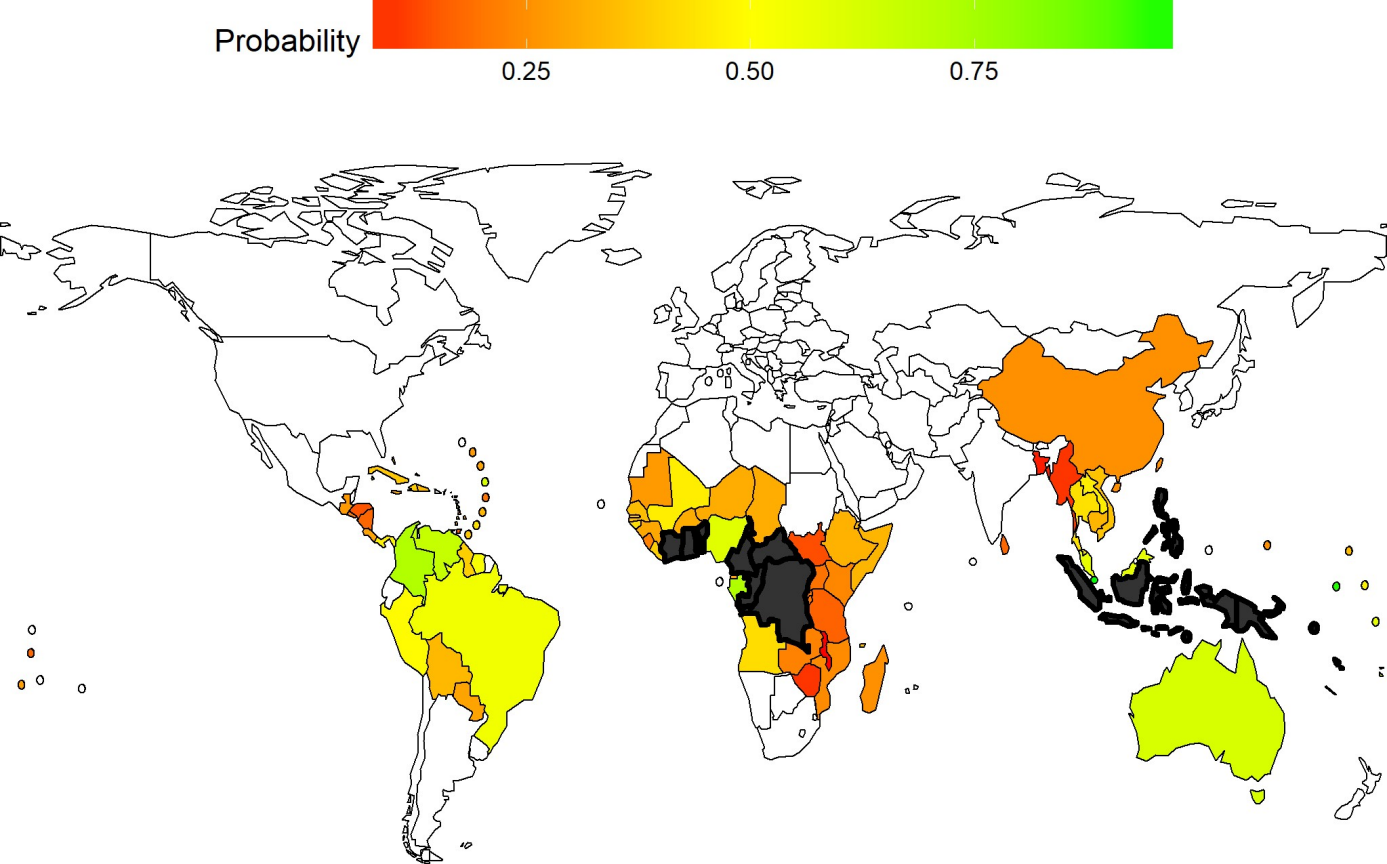
Predicted probability of case reporting in previously endemic countries (current status unknown) conditional on ongoing transmission, three year period ending 2015. Currently endemic countries (current status known) are depicted in grey. In addition to Member States, there are 9 countries or areas with a history of yaws for which data are not included in this map: British Virgin Islands, Guadeloupe, French Guiana, Guam, Montserrat, Martinique, New Caledonia, Puerto Rico, and Wallis and Futuna. Created using R and World Health Organization shapefiles under Creative Commons license (CC-BY).

Using Model (3), we predicted the probability that a given country would report cases in the three year period ending in 2015, through passive surveillance alone, if there were in fact new cases (i.e., conditional on there being ongoing transmission). The predicted probabilities ${\hat{REP3}}_{i2015}$ are displayed in Fig 4 for 86 previously endemic countries (current status unknown). Since we used a random effects model, we were able to predict probabilities also for the 7 Member States with only non-specific case reports (Bangladesh, El Salvador, Honduras, Marshall Islands, Myanmar, Nauru and Nicaragua), with the conservative assumption that a single case was reported in 1945.

Predicted probabilities and their 95% confidence intervals are presented, by country, in Supporting Information **S2 Table**.

There are 66 countries and areas with less than a 50% probability of reporting cases in the three year period ending 2015, even if there had in fact been ongoing transmission. That leaves only 20 countries and areas with a more than 50% probability of reporting cases in the absence of active surveillance. If we consider uncertainty and take the lower bound of the 95% confidence interval, only 8 countries and areas had a better than 50:50 chance of reporting cases. Only four of these countries/areas have a probability (best estimate) of 80% or higher: Puerto Rico, Guadeloupe, Nauru and Singapore.

The median predicted probability for currently endemic countries and areas is 73%, ranging from 6% in Wallis and Futuna Islands to 89% in Congo. Excluding Wallis and Futuna, which is an outlier (with a 100% rural population), the range is 60–89%. The prediction that the probability of reporting is significantly less than 100% even for currently endemic countries is given credence by the fact that WHO has not received reports from these countries in all years since formal adoption of the yaws eradication target.

## Discussion

There is a need to prioritize countries with a history of yaws so that international resources for global eradication can be deployed efficiently.

So far the focus has been on mass treatment in the Member States that are currently reporting cases to WHO. A strategy for roll-out of mass treatment for yaws has been articulated [9]. WHO continues to focus on mobilizing the necessary resources, including donations of medicines and rapid diagnostic tests, for “total community treatment” of endemic villages. Based on our review of the literature, there is a territory (Wallis and Fortuna Islands) belonging to a Member State (France) that can also be considered currently endemic, but from which WHO is not currently receiving reports.

It is also important to develop a strategy for surveillance in the 86 previously endemic countries whose current status is unknown. Within this large and diverse group, we have identified a group of 20 countries with more than a 50% probability of reporting cases in the absence of active surveillance. These countries could, in principle, begin to prepare a dossier for certification. The dossier would have to include, based on current requirements, no evidence of clinical yaws among children aged 0–15 years and evidence of the absence of rapid plasma reagin sero-reactivity among children aged 1–5 years [9].

For subsequent certification, as in the case of guinea worm disease eradication [11], countries should be required to provide WHO a signed declaration confirming the absence of local transmission and also to complete an assessment of whether they indeed have satisfactory surveillance which could detect yaws cases if they occurred. Quality standards for “satisfactory surveillance” need to be formalized and documented, based on the experience of the expert group led by WHO that certified the eradication of yaws in India in 2016.

The remaining 66 previously endemic countries will likely need international support for active surveillance. The ones with a high probability of transmission should undertake population surveys (they will require mapping for eventual intervention); others, with a low probability of transmission, should consider purposive case search (to provide evidence of the absence of cases). In this study, we cannot distinguish between these two groups of countries because we have only estimated the probability of reporting conditional on transmission, not the probability of transmission itself. The latter is hardly possible with the available data, but a Delphi approach could perhaps permit meaningful grouping of these countries.

There are several other limitations to this study.

First, the literature identified 235 studies for which the abstract or full-text could not be retrieved. However, when the title or abstract referred to yaws in a specific country, we confirmed that case reports had been extracted from other references. Most (141 of 235) of the studies in this category were published in 1960 or earlier–during a time when reporting to WHO was still quite complete–so it is likely that relevant data were available to us from other sources.

Second, more troublingly, is the inconsistent definition of cases in the literature–sometimes referring to clinically suspected, sometimes lab-confirmed clinical cases; sometimes officially notified, sometimes independently reported cases; sometimes infectious cases only, sometimes both infectious and non-infectious cases. On the other hand, we have used a measure of case reporting that should be relatively insensitive to inconsistencies in case definitions: we used not the number of cases reported, but simply a binary variable indicating that there was at least one case reported.

Third, the descriptive model was limited by the availability of historical data from 1945. For example, we could not include poverty or income inequality measures because data were available only from 1980, and then only somewhat inconsistently until 1990. In high income countries where economic wealth is unequally distributed over geographic areas, there may remain neglected communities with inadequate surveillance.

Fourth, we limited our regression analysis to modelling presence/absence of case reporting conditional on ongoing transmission in the years before the last reported case. A model of disease transmission in the years after the last reported case was beyond the scope of this paper.

Fifth, the 50% cut-off was chosen arbitrarily. Our recommendations are therefore based primarily on the relative ranking of countries, not on their predicted probabilities, which are low overall. Threshold probabilities can be made more evidence-based as active surveillance is undertaken. One might wish to sample some of the countries with a high probability of reporting but no reports and do active surveillance regardless; if cases are found, there is reason to question the robustness of the model and/or cut-off.

In spite of these limitations, our work will facilitate discussions with countries to assess their interest in and readiness for either certification (based on passive surveillance) or active surveillance. Integration of active surveillance with other large scale prevalence surveys for trachoma and other neglected tropical diseases could be considered [28–30].

## Supporting information

S1 Fig Flow diagram of literature review. (DOCX)

S1 Table Studies from which data were extracted (ordered by year of publication). (DOCX)

S2 Table Predicted probability of case reporting and 95% confidence interval, by country. (DOCX)
