# Supplementary material for: Prioritizing surveillance activities for certification of yaws eradication based on a review and model of historical case reporting
Source: PLoS Negl Trop Dis. 2018 Dec 4;12(12):e0006953. doi: 10.1371/journal.pntd.0006953 (PMC6294396; doi:10.1371/journal.pntd.0006953)
Supplement: S1 Fig — (DOCX) [file pntd.0006953.s001.docx]

No active clinical non-imported yaws cases reported

(n = 1744)

Full-texts not available when necessary
(n = 73)

Abstracts and full-texts not available (n = 162)

Additional records identified through other sources
(n = 210)

Records identified through Pubmed, Global Health – CABI and Global Index Medicus
(n = 2434)

Abstracts or full-texts from which data were extracted
(n = 413)

Records after duplicates removed
(n = 2392)

Abstracts assessed
(n = 2230)

Full-texts assessed if necessary

(n = 2157)
