# Supplementary material for: Prioritizing surveillance activities for certification of yaws eradication based on a review and model of historical case reporting
Source: PLoS Negl Trop Dis. 2018 Dec 4;12(12):e0006953. doi: 10.1371/journal.pntd.0006953 (PMC6294396; doi:10.1371/journal.pntd.0006953)
Supplement: S1 Table — (DOCX) [file pntd.0006953.s002.docx]

1.

BINNS RT. A Study of Diseases in Australian Natives in the Northern Territory. Medical Journal of Australia. 1945;1: 421–6.

2.

GUIMAEAES FN. Penicillin in the Treatment of Tertiary Yaws. Brasil-Medico. 1945;59: 89–91.

3.

GUIMARAES FN. The Curative Action in Yaws of Penicillin taken by Mouth. Hospital (Rio de Janeiro, Brazil). 1945;28: 229–32.

4.

IBARRA PEREZ R, GONZALEZ PRENDES MA. Yaws in Bayamo (Cuba). Revista de Sifilografia, Leprologia y Dermatologia. 1945;2: 206–18.

5.

LOPES CF, LAENDER JF. Montenegro Skin Test in Muco-Gutaneous Leishmaniasis. Negative for Yaws. Brasil-Medico. 1945;59: 41–6.

6.

STAVAUX R. Two Cases of Yaws with Bilateral Perioribital Hyperostosis. Rec Tmvattx Set Med Congo Beige. 1945; 177–81.

7.

TOMPSETT RR, KAUER GL. Penicillin Treatment of Early Yaws. American Journal of Tropical Medicine. 1945;25: 275–6.

8.

WHITEHILL R, AUSTRIAN R. Further Observation on the Treatment of Yaws with Penicillin. Bulletin of the Johns Hopkins Hospital. 1945;76: 274–94.

9.

HACKETT CJ. The Clinical Course of Yaws in Lango, Uganda. Transactions of the Royal Society of Tropical Medicine and Hygiene. 1946;40: 206–17. doi:10.1016/0035-9203(46)90067-3

10.

MAUZE J. Contribution to the Study of Malaria in the New Hebrides, chiefly during the War in the South-West Pacific, January 1942 to December 1943. Medecine Tropicale. 1946;6: 109–38.

11.

ARJE SL. Yaws treated with Single Massive Doses of Penicillin. Naval Medical Bulletin. 1947;47: 965–9.

12.

CHINN M. A New Study of Wassermann and Kahn Reactions in Malaria. Ann Soc Belge de Med Trop. 1947;27: 5–15.

13.

DE ALBUQUERQUE AFR, MACHADO AC. Disease in Alagoas. Rev Hig e Satide Publica. 1947;5: 21–34.

14.

DWINELLE JH, SHELDON AJ, REIN CR, STERNBERG TH. Evaluation of Penicillin in the Treatment of Yaws. Final Report. American Journal of Tropical Medicine. 1947;27: 633–41.

15.

DWINELLE JH. Penicillin in the Treatment of Yaws. Kuba. 1947;3: 84–6.

16.

GONÇALVES AP. Results of Inoculation of the Spiroehaeta of Pinta into Yaws Patients. Hospital (Rio de Janeiro, Brazil). 1947;31: 83-8; 91–3.

17.

GUIMARÄES FN. Attempted Prophylaxis of Yaws by Mass Treatment with Penicillin in a Riverine District. 1947. pp. 301–7. Available: https://www.cabdirect.org/cabdirect/abstract/19502902010

18.

GUIMARÄES FN. Dyschromic Yaws Lesions Simulating Pinta. Brasil-Medico. 1947;61: 81–7.

19.

HACKETT CJ. Incidence of Yaws and of Venereal Diseases in Lango (Uganda). British Medical Journal. 1947;40: 88–90.

20.

APTED I, HARDING RD, GOSDEN M. A Clinical and Serological Follow-up of Yaws Cases treated by Acetylarsan and Bismuth Sodium Potassium Tartrate. Transactions of the Royal Society of Tropical Medicine and Hygiene. 1948;42: 55–64.

21.

COOK MJ. Neurosyphilis in the Tropics. Journal of Venereal Disease Information. 1948;29: 204–7.

22.

SCHERPENHUYSEN AA. Bismuth in the Treatment of Yaws. Nederlands Tidjschrift voor Geneeskunde. 1948; 2741–9.

23.

Antunes PCA. Program for the eradication of yaws from Haiti. World Health Organization; 1949.

24.

DE BRAUWERE P, CONGO BELGE. Report of the Activities of Foréami during 1946 and 1947. [Internet]. Brussels: 112 Rue du Commerce.; 1949 p. 140 pp. Available: https://www.cabdirect.org/cabdirect/abstract/19492901509

25.

FERREIRA FSDC, PINTO AR. S.T.B. in the Treatment of Yaws. Anais do Instituto de Medicina Tropical. 1949;6: 115–26.

26.

FRIEDHEIM EAH. A Five Day Pérorai Treatment of Yaws with STB, a New Trivalent Arsenical. American Journal of Tropical Medicine. 1949;29: 185–8.

27.

REIN CR. Treatment of yaws in the Haitian peasant. J Natl Med Assoc. 1949;41: 60–65.

28.

SEPULVEDA G Jr, IBARRA LM. A Critical Study of the Quantitativ" Kahn Test (Blood) In 100 Cases of Yaws. Journal of the Philippine Islands Medical Association. 1949;25: 487–9.

29.

AMPOFO O, FINDLAY GM. Chloramphenicol in the Treatment of Yaws and Tropical Ulcer. Transactions of the Royal Society of Tropical Medicine and Hygiene. 1950;44: 315–18. doi:10.1016/0035-9203(50)90059-9

30.

AMPOFO O, FINDLAY GM. The Treatment of Yaws by Aureomycin. Transactions of the Royal Society of Tropical Medicine and Hygiene. 1950;44: 311–13. doi:10.1016/0035-9203(50)90058-7

31.

DE AQUINO UM. Fixation of Complement and Flocculation (with Cardiolipin) in Sérum and Cerebrospinal Fluid in Yaws. Brasil-Medico. 1950;64: 256–8.

32.

FERREIRA FC, PINTO AR. Treatment of Yaws with an Acetarsol Derivative. Bulletin de la Société de Pathologie Exotique. 1950;43: 33–7.

33.

GOMES L de S. Laboratory Findings in Two Cases of Framboesia. Circular Form of Treponema pertenue. Revista do Instituto Adolfo Lutz. 1950;10: 67–70.

34.

GUIMARÄES FN, TRAVASSOS J. Effect of Terramycin on Yaws. Hospital (Rio de Janeiro, Brazil). 1950;38: 295–9.

35.

GUIMARÃES FN. The Clinical Development of Yaws in Baixada Fluminense State of Rio de Janeiro, Brazil. Puerto Rico Journal of Public Health and Tropical Medicine. 1950;26: 1–34.

36.

GUIMARES FN. Procaine Penicillin in. Large Doses in the Treatment of Yaws. Hospital (Rio de Janeiro, Brazil). 1950;37: 361–9.

37.

MARPLES MJ. The Incidence of certain Skin Diseases in Western Samoa : a Preliminary Survey. Transactions of the Royal Society of Tropical Medicine and Hygiene. 1950;44: 319–32. doi:10.1016/0035-9203(50)90060-5

38.

MONTEL LR, LEGAC P, MULET M. Trials of Chaulmoogra Oil in the Treatment of Yaws. Bulletin de la Société de Pathologie Exotique. 1950;43: 681–4.

39.

REIN CR, KITCHEN DK, PETRUS EA. Repository Penicillin Therapy of Yaws in the Haitian Peasant. A Clinical and Serologie Survey. Journal of Investigative Dermatology. 1950;14: 239–45.

40.

TAVARES A. Outpatient Treatment of Yaws with Procaine-Pemeillin G. Medicina, Cirurgia, Farmacia. 1950; 418–26.

41.

ABREU ARREDONDO C. The Use of Penicillin in the Yaws Campaign in Venezuela. Boletín de la Oficina Sanitaria Panamericana. 1951;31: 365–7.

42.

AMPOFO O, FINDLAY GM. Terramycin in the Treatment of Yaws and Tropical Ulcer. Transactions of the Royal Society of Tropical Medicine and Hygiene. 1951;45: 261–3. doi:10.1016/S0035-9203(51)90965-0

43.

ANGULO JJ, WATSON JHL, WEDDERBURN CC, LEÓN-BLANCO F, VÁRELA G. Electronmicrography of Treponemas from Cases of Yaws, Pinta, and the so-called Cuban Form of Pinta. American Journal of Tropical Medicine. 1951;31: 458–78.

44.

APTED I. Bismuth Salieylate in the Treatment of Yaws. Transactions of the Royal Society of Tropical Medicine and Hygiene. 1951;45: 389–92. doi:10.1016/S0035-9203(51)80010-5

45.

CONGO BELGE DG des SM. Annual Report of the Medical Directorate-General. [Internet]. 1951 p. 89 pp. Available: https://www.cabdirect.org/cabdirect/abstract/19522900603

46.

DA SILVA AC. Health and Economic Problems in Amazonia. Folha Medica. 1951;32: 4–8.

47.

FAINE S, HERCUS CE. Infections in Rarotonga, Cook Islands. Transactions of the Royal Society of Tropical Medicine and Hygiene. 1951;45: 341–52. doi:10.1016/S0035-9203(51)80005-1

48.

FERREIRA FS da C. Yaws and Its Treatment in Portuguese Guinea. Gazeta Medica Portuguesa. 1951;4: 1034–45.

49.

GUTHE T, REYNOLDS F. World Health and Treponematoses. Brit J Venereal Dis. 1951;27: 1–19.

50.

HILL KR, RHODES K, ESCOFFERY GS, MURRAY CC. Framboesia (Yaws) treated with Aureomycin. West Indian Medical Journal. 1951;1: 93–6.

51.

LOUGHIN EH, JOSEPH A, SCHAEFFER K. Aureomyein in the Treatment of Yaws. American Journal of Tropical Medicine. 1951;31: 20–23.

52.

LOUGHLIN EH, JOSEPH A, SCHAEFFER K. Phase Contrast Microscopy for Demonstration of Treponema pertenue in Yaws Lesions. American Journal of Tropical Medicine. 1951;31: 26.

53.

LOUGHLIN EH, JOSEPH AA. Terramycin in the Treatment of Yaws. Antibiotics & Chemotherapy. 1951;1: 76–87.

54.

MONTEL LR, LE GAC P, MULET M. New Cases of Yaws treated with Chaulmoogra OIL. Bulletin de la Société de Pathologie Exotique. 1951;44: 173–4.

55.

MONTEL LR. Antibiotic Action of Chaulmoogra Oil. Its Action in Treponematoses. Revue Coloniale de Medecine et de Chirurgie. 1951;23: 232–4.

56.

ESPINOSA A, AMADOR GUEVARA J. Control of Yaws in the Republic of Panama. Archivos Med  Panatnefos. 1952;1: 8–27.

57.

JELLIFFE DB, HUMPHREYS J. Lesions of the Feet in African Soldiers. (A Clinical Survey of 464 Nigerian Troops.). Journal of Tropical Medicine and Hygiene. 1952;55: 1–5.

58.

LEVITAN S, DURAND JB, PETRUS E, JACOBS JC. Treatment of Yaws by a Single Dose of Penicillin. Boletín de la Oficina Sanitaria Panamericana. 1952;33: 565–81.

59.

Pan American Sanitary Organization. Annual report of the Director of the PASB, 1951 [Internet]. Washington, D.C; Pan American Sanitary Bureau; 1952. Available: http://apps.who.int/iris/handle/10665/169449

60.

PINTO AR, RONCON R. Stovarsol in the Treatment of Yaws. Boletim Cultural da Guine Portuguesa. 1952; 249–54.

61.

RAMANARAO PV. Studies in Yaws (Koya Rogam). A Review of 1,000 Gases of Yaws from Warangal District, Hyderabad State (Deooan). Indian Medical Gazette. 1952;87: 437–44.

62.

REYNOLDS FW, GUTHE T. Treponemal Disease Control in Underdeveloped Countries: Experiences in Mass Therapy. American Journal of Syphilis. 1952;36: 424–32.

63.

TRATMAN EK. A Case of Advanced Tertiary Yaws. Brit Dent J. 1952;92: 70–71.

64.

BLOMFIELD D. Observations upon Yaws and its Treatment in Embu District. East African Medical Journal. 1953;30: 323–32.

65.

CONGO BELGE. Annual Report of the Medical Directorate General, 1952. [Internet]. 1953 p. 107 pp. Available: https://www.cabdirect.org/cabdirect/abstract/19542900440

66.

CRUZ AH. Integration of yaws control into the permanent health structure of the Philippines. Bull World Health Organ. 1953;8: 345-53; discussion 365-368.

67.

DE ALMEIDA JO, PESSOA SB, COVKLLO A. Quantitative ComplementFixation Reaction in Yaws. Hospital (Rio de Janeiro, Brazil). 1953;43: 373–80.

68.

DE AQUINO UM. Intradermal Tests in Yaws. Hospital (Rio de Janeiro, Brazil). 1953;43: 765–75.

69.

DELGADO JADBA. Endemic Disease and the Causes of Malnutrition in Tete (Mozambique). Anais do Instituto de Medicina Tropical. 1953;10: 1779–92.

70.

DE NORONIIA C. Treponematosis In Portuguese Territories Overseas. Rev Portuguosa do Med Militar. 1953;1: 49–66.

71.

DRICOT C, CONGO BELGE F. Report of the Activities of Foréami during 1952. [Internet]. Brussels: 39, Rue du Commerce; 1953 p. 118 pp. Available: https://www.cabdirect.org/cabdirect/abstract/19542901588

72.

FERREIRA EC. Distribution and Incidence of some of the Diseases endemic in Angola. Anais do Instituto de Medicina Tropical. 1953;10: 1739–75.

73.

GUTHE T, REYNOLDS FW, KRAG P, WILLCOX RR. Mass Treatment of Treponemal Diseases, with particular reference to Syphilis and Yaws. British Medical Journal. 1953; 594–8.

74.

Hackett CJ. Consolidation phase of yaws control: Experiences in Africa. Bull World Health Organ. 1953;8: 297–343.

75.

Hackett CJ. Extent and nature of the yaws problem in Africa. Bull World Health Organ. 1953;8: 129-182; discussion 205-210.

76.

HACKETT CJ. On the Epidemiology and Prevention of Yaws. Rev Bmsileim Malariologia. 1953;5: 5–9.

77.

KEENY SM. The role of the United Nations International Children’s Emergency Fund in yaws control. Bull World Health Organ. 1953;8: 379–392.

78.

KETTERER WA. Village Polycllnics in Middle Java. Public Health Reports. 1953;68: 558–62. doi:10.2307/4588481

79.

MARPLES MJ, BACON DF. I. Observation on Yaws and certain Skin Diseases in Manono, Western Samoa. Transactions of the Royal Society of Tropical Medicine and Hygiene. 1953;47: 141–7. doi:10.1016/0035-9203(53)90067-4

80.

NERY GUIMARAES F. Yaws in Brazil. Bull World Health Organ. 1953;8: 225–242.

81.

PETRUS E, LEVITAN S, PAOLIELLO A, NICOL R. [The anti-yaws campaign in Haiti]. Bull World Health Organ. 1953;8: 261-71; discussion 293-295.

82.

ROCK EE. Yaws on Guam : Treatment and Environmental Considerations. American Journal of Tropical Medicine and Hygiene. 1953;2: 74–8.

83.

SCHMID EE, VELAUDAPILLAI T. Meinicke (Kvittingen) and Mueller Ballung (Clotting) Tests in comparison with Standard Kahn and Cardiolipin (Kline) Tests. Ceylon Journal of Medical Science. 1953;8: 157–60.

84.

SEPULVEDA G Jr, IBARRA LM. The Effectiveness of Penicillin in the Treatment of Yaws (Primary and Secondary). Journal of the Philippine Islands Medical Association. 1953;29: 727–33.

85.

SOETOPO M, WASITO R. Experience with yaws control in Indonesia; preliminary results with a simplified  approach. Bull World Health Organ. 1953;8: 273–295.

86.

VAUCEL MA. [Yaws in the French African territories]. Bull World Health Organ. 1953;8: 183–210.

87.

VENEREAL DISEASE RESEARCH INSTITUTE INDONESIA-SOURABAYA., editor. The Framboesia Campaign in Indonesia. [Internet]. 1953. Available: https://www.cabdirect.org/cabdirect/abstract/19542901460

88.

WHO. First International Symposium on Yaws Control [Internet]. Geneva: World Health Organization; 1953. Report No.: 15. Available: http://apps.who.int/iris/bitstream/10665/41140/1/WHO_MONO_15.pdf

89.

WHO. The yaws programme assisted by UNICEF and WHO: a progress report by WHO on projects in Haiti, Indonesia, Thailand and the Philippines. World Health Organization; 1953.

90.

WHO. Yaws: A progress report by WHO on projects in Haiti, Indonesia, Thailand and the Phillipines. Geneva: World Health Organization; 1953.

91.

DE AQUINO UM. Intradermal Test in Yaws. II. Nature of the Reaction and its Specificity. Hospital (Rio de Janeiro, Brazil). 1954;45: 451–462.

92.

FURTADO TA, DE ALMEIDA AF. [Frambesai tropica (bouba) in the northeast of Minais Gerais; analysis of 5,385 cases]. An Bras Derm Sifilogr. 1954;29: 1–11.

93.

GUTHE T, WILLCOX RR. Treponematoses: a World Problem. Chronicle of the World Health Organization. 1954;8: 37–113.

94.

Huggins D. Report on Mission to Fiji November-December 1953 with Special Reference to Yaws Control. World Health Organization; 1954.

95.

Huggins D. Report on  Mission to Western Samoa November 1953 with Special Reference to Yaws Control. World Health Organization; 1954.

96.

LAPEYSSONNIE L. [English title not available]. Annales de Dermatologie et de Syphiligraphie. 1954;81: 644–651.

97.

LAPEYSSONNIE L. The Behaviour of Classical Serum Reactions in Africans. (Sera from Normal Persons and Cases of Yaws, Leprosy and Trypanosomiasis). Ann Biol clin. 1954;12: 1–14.

98.

LE GAC P, SICÉ A, VIOLLIER G. Determination by Electrophoresis of the Proteins of Blood Serum of Africans from Oubangui in Health and Disease. Bulletin de la Société de Pathologie Exotique. 1954;47: 108–12.

99.

LOUGHLIN EH, JOSEPH AA, DUVALIER F. Oxytetracycline Intramuscular in the Treatment of Yaws (Pian). Antibiotics & Chemotherapy. 1954;4: 155–64.

100.

MEDINA R, ABREU A., C. Campaign against Yaws in Venezuela. Revista de Sanidad y Asistencia Social. 1954;19: 345–59.

101.

MEDINA R. Reactions produced by Inoculation of Treponema pertenue in Pinta, Yaws and Syphilis and its Application to Test of Cure. Archivos Venezolanos de Patologia Tropical y Parasitologia Medica. 1954;2: 51–87.

102.

MILLS AB. A Malaria Survey of Futuna in the New Hebrides. Journal of Tropical Medicine and Hygiene. 1954;57: 99–107 pp.

103.

AMERICAN GEOGRAPHICAL SOC., editor. World Distribution of Spirochetal Diseases. I. Yaws, Pinta, Bejel. Atlas of Diseases. 1955; Available: https://www.cabdirect.org/cabdirect/abstract/19552901902

104.

BELL S. The Amera People of Kenya. A Medical and Social Study. Part I: Geographical and Ethnological Background. Journal of Tropical Medicine and Hygiene. 1955;58: 223–39.

105.

DE BEAUX J. Yaws In the Western Solomon Islands. Journal of Tropical Medicine and Hygiene. 1955;58: 33–8.

106.

DE BEAUX J. Yaws in the Western Solomon Islands. Second Resurvey of the Island of Simbo in March 1955. Journal of Tropical Medicine and Hygiene. 1955;58: 136–7.

107.

D’MELLO JMF, KRAG P. Serological Studies of Yaws in Thailand. Bulletin of the World Health Organization. 1955;13: 1003–40.

108.

EDESON JFB. Clinical Diagnosis of Filariasis. Transactions of the Royal Society of Tropical Medicine and Hygiene. 1955;49: 488–9. doi:10.1016/0035-9203(55)90018-3

109.

GHESTIN F. Treatment of Yaws with Benzathine Penicillin. Bulletin de la Société de Pathologie Exotique. 1955;48: 643–9.

110.

Hackett CJ. Report on yaws in Sierrra Leone. World Health Organization; 1955.

111.

LAPEYSSONNIE L. How to interpret Serum Tests for Treponematoses among Africans. Acta Tropica. 1955;12: 29–37.

112.

LI H, SOEBEKTI E. Serological Study of Yaws in Java. Bulletin of the World Health Organization. 1955;12: 905–43.

113.

MASSEGUIN A, BRUMPT V, DULAC P. The Treatment of Treponematoses in Tropical Africa by the Salts of Diamine-Penicillin. Bulletin de la Société de Pathologie Exotique. 1955;48: 422–9.

114.

WHO. Regional Office of the Western Pacific. Stories of the World Health Organization for the Western Pacific [Internet]. Manila : WHO Regional Office for the Western Pacific; 1955. Available: file:///C:/Users/nyobd/Downloads/Stories_wpro_eng.pdf

115.

AUGÉRE R, LACOUR M. The Treponematoses in New Caledonia. An Epidemiological and Serological Investigation. Medecine Tropicale. 1956;16: 497–513.

116.

BELL S. The Amera People of Kenya. A Medical and Social Study. Part V: Treponematosis. Journal of Tropical Medicine and Hygiene. 1956; 31–40.

117.

DA CRUZ-FERREIRA FS, STEEENBERG H. Some aspects of Yaws in Liberia. American Journal of Tropical Medicine and Hygiene. 1956;5: 1036–50.

118.

Klokke AH. Yaws in the households of Tjawas (Central Java): an epidemiologic study from the Treponematoses Control Program in Indonesia. Universitas Gadjah Mada at Jogjakarta. 1956.

119.

KRANENDONK O. The Value of the Chediak YDRL Test in Yaws Serology compared with the Standard VDRL-Slide Test. Documenta de Medicina Geographica et Tropica. 1956;8: 365–74.

120.

MARROQUIN J. Distribution and Incidence of Yaws in Peru. Rev, Peruana de Salud Publica. 1956;5: 205–210.

121.

Pan American Health Organization. Summary of four-year reports on health conditions in the Americas 1950-1953 [Internet]. Washington, D.C: Organizacion Panamericana de la Salud; 1956. Available: http://iris.paho.org/xmlui/handle/123456789/1298

122.

PINTO AR da C. Activities of the Mission for the Study and Control of Sleeping Siokness in Portuguese Guinea during the Year 1955. Anais do Instituto de Medicina Tropical. 1956;13: 275–31.

123.

RAMOS SF. Venereal Diseases in the Dermato-Venereoíogical Department of the Central Miguel Bombarda Hospital at Lourenço Marques. Anais do Instituto de Medicina Tropical. 1956;13: 487–505.

124.

SAMAME GE. Treponematosis Eradication, with special reference to Yaws Eradication in Haiti. Bulletin of the World Health Organization. 1956;15: 897–910.

125.

SOETOPO M, WASITO R, SOEDARSONO H, TJOKRODIPO D. The Indonesian Treponematoses Control Project. Bulletin of the World Health Organization. 1956;15: 937–58.

126.

WHO. Plan of Operations For an Expanded Treponematoses Project in St. Vincent and its Dependencies. World Health Organization; 1956.

127.

ZAHRA A. Yaws eradication campaign in Nsukka Division, Eastern Nigeria. Bull World Health Organ. 1956;15: 911–935.

128.

DAVIDSON WS. Health and Nutrition of Warburton Range Natives of Central Australia. Medical Journal of Australia. 1957;2: 601–5.

129.

FAWKES MA. A Short History of Yaws in Trinidad. West Indian Medical Journal. 1957;6: 189–204.

130.

FURTADO TA. Late Manifestations of Yaws. Arch Dermat. 1957;76: 446–51.

131.

GENTLE GHK. The Significance of Asymptomatic Serum-Positivity in Trinidad and Tobago with special reference to Yaws. West Indian Medical Journal. 1957;6: 217–24.

132.

HUME JC, FACIO G. Analysis of the Results of treating Yaws with Single Injections of Procaine Penicillin in Two Per Cent Aluminium Monostearate. Boletín de la Oficina Sanitaria Panamericana. 1957;42: 31–53.

133.

MCFADZEAN JA, MCCOURT JF, WILKINSON AE. Treponematoses in Gambia, West Africa. Transactions of the Royal Society of Tropical Medicine and Hygiene. 1957;51: 169–81. doi:10.1016/0035-9203(57)90062-7

134.

MCLETCHIE JL. Report of Second International Conference on Control of Yaws: Nigeria, 1955. Journal of Tropical Medicine and Hygiene. 1957;60: 27-38; 62-73.

135.

Organización Panamericana de la Salud. Seminario sobre erradicación de treponematosis. Puerto Principe, Haití, Otubre 1956 [Internet]. Organización Panamericana de la Salud Washington, D.C; 1957. Available: http://apps.who.int/iris/bitstream/10665/170187/1/42168.pdf

136.

PETRUS E, VELARDE THOMÉ J. Five Years of the Campaign against Yaws in Haiti. Boletín de la Oficina Sanitaria Panamericana. 1957;42: 22–30.

137.

TALLACK EJK. Epidemiology of Yaws in Zanzibar and Pemba. East African Medical Journal. 1957;34: 533–43.

138.

VAN DER HOFF NM. Two Yaws Endemics in South-West Borneo. Documenta de Medicina Geographica et Tropica. 1957;9: 281–90.

139.

WHO. Meeting for the Coordination of Mass Yaws Campaigns in West and Central Africa. Geneva: World Health Organization; 1957.

140.

WHO. Tripartite Plan of Operations for an Expanded Treponematoses Project in St. Lucia. World Health Organization; 1957.

141.

WHO. Tripartite Plan of Operations for a Treponematoses Project in Trinidad and Tobago. World Health Organization; 1957.

142.

YU K-Y. Frambesia. A Study of 125 Cases. Chinese Medical Journal. 1957;75: 616–25.

143.

GUIRAUD R. Placental Therapy in Tropical Conditions. ed Trop Marseilles. 1958;18: 98–100.

144.

Huggins D. Report on Field Visit to Fiji and Sydney 3-11 August 1957 and 1-8 October 1957. World Health Organization; 1958.

145.

Kranendonk OJM. Serological and epidemiological aspects in yaws control : report on a mass treatment campaign against yaws in Netherlands New Guinea. University of Amsterdam. 1958.

146.

Organización Panamericana de la Salud. Reported cases of notifiable diseases in the Americas 1946-1955 [Internet]. Washington, D.C: Organizacion Panamericana de la Salud; 1958. Report No.: Publicación Científica 37. Available: http://apps.who.int/iris/handle/10665/170194

147.

Pan American Health Organization. Summary of four-year reports on health conditions in the Americas [Internet]. Washington, D.C: Organizacion Panamericana de la Salud; 1958. Available: http://iris.paho.org/xmlui/handle/123456789/1322

148.

POULSON NH. The Clinical Interpretation of Serological Reactions in Venereal and Endemic Treponematosis. American Journal of Public Health. 1958;48: 1622–7. doi:10.2105/AJPH.48.12.1622

149.

PRATES M. Epidemiology of Yaws in the Province of Mozambique. Anais do Instituto de Medicina Tropical. 1958;15: 359–82.

150.

VAN ZANEN GE. Bone Yaws. Report on a Case. Tropical and Geographical Medicine. 1958;10: 354–62.

151.

WHO. Plan of Operations for Expanding the Yaws Control Campaign in Sierra Leone. World Health Organization; 1958.

152.

COBBAN K. McL. Analysis of a Year’s Outpatients at University College Hospital, Ibadan. Journal of Tropical Medicine and Hygiene. 1959;62: 129–34.

153.

ENGELHARDT HK. A Study of Yaws (Does Congenital Yaws Occur?). Journal of Tropical Medicine and Hygiene. 1959;62: 238–40.

154.

MEDINA R. Evolutive Serological Responses in Yaws Patients. Archivos Venezolanos de Patologia Tropical y Parasitologia Medica. 1959;3: 290–97.

155.

RELVICH AL. Observations on the Rate of Resolution of Yaws Lesions after Procaine Benzyl Penicillin Treatment. Transactions of the Royal Society of Tropical Medicine and Hygiene. 1959;53: 75–7. doi:10.1016/0035-9203(59)90087-2

156.

RELVICH AL. Yaws as a Factor in the Causation of the Contractures of Fingers among the Population of West Africa. West African Medical Journal. 1959; 78–80.

157.

WHO. First Inter-country Yaws Control Co-ordination Meeting, Kuala Lumpur, 13-18 April 1959 [Internet]. Manila: World Health Organization Regional Office for the Western Pacific; 1959. Available: http://iris.wpro.who.int/bitstream/handle/10665.1/1952/WPRO_0078_eng.pdf

158.

World Health Organization. First report on the world health situation 1954-1956 [Internet]. Geneva: World Health Organization; 1959. Report No.: 94. Available: http://apps.who.int/iris/handle/10665/85718

159.

World Health Organization. Regional Office for the Western Pacific. The First Inter-country Yaws Control Co-ordination Meeting, Kuala Lumpur, 13-18 April 1959 : final report [Internet]. Manila : WHO Regional Office for the Western Pacific; 1959. Available: http://apps.who.int/iris/bitstream/10665/207120/1/WPRO_0078_eng.pdf

160.

FRY EI. Health Survey of Children from Rarotonga, Cook Islands. (1) Medical Examination and Evaluation. Journal of Tropical Pediatrics. 1960;5: 121–9.

161.

GUTHE T. The Treponematoses as a World Problem. Brit J Venereal Dis. 1960;36: 67–77.

162.

MARPLES MJ, MARPLES RR, MUIR B. Microbiological Studies in Western Samoa. I. Effects of a Yaws Control Programme on the Incidence of Superficial Skin Lesions and their Aerobic Bacterial Flora, with an Appendix on the Normal Flora of the Nose. Transactions of the Royal Society of Tropical Medicine and Hygiene. 1960;54: 155–65. doi:10.1016/0035-9203(60)90052-3

163.

MONTES DE OCA I, MAEKELT GA. Use of Antigens Prepared from Cultures of Reiter’s Tréponème in Cases of Yaws, Pinta and Syphilis. Archivos Venezolanos de Med Tropical y Parasitologia Medica. 1960;3: 161–72.

164.

Organización Panamericana de la Salud. Reported cases of notifiable diseases in the Americas 1949-1958 [Internet]. Washington, D.C: Organizacion Panamericana de la Salud; 1960. Report No.: Publicación Científica 48. Available: http://apps.who.int/iris/handle/10665/170206

165.

Roberts L. Report on Field Visit to the Kingdom of Tonga 25-28 February 1960. World Health Organization; 1960.

166.

Tross DF. Assignment Report on the Yaws “Training Survey”: Cook Islands. World Health Organization; 1960.

167.

World Health Assembly, 13. Committee on Programme and Budget - provisional minutes of the sixth meeting, Palais des Nations, Geneva, Wednesday, 11 May 1960, at 9.30 a.m. [Internet]. 1960. Available: http://apps.who.int/iris/bitstream/10665/134356/1/WHA13_PB-Min-6_eng.pdf

168.

BROWNE SG. Juxta-Articular Nodules in Yaws: a Clinical Study of 210 Cases. Ann Trop Med Parasit. 1961;55: 309–313.

169.

CRUZ AH, SUVA JP, JUSTINIANO GSD. The Yaws Control Programme in the Philippines with WHO/UNICEF Assistance. Journal of the Philippine Islands Medical Association. 1961;37: 675–82.

170.

DEENY J. Health Survey of the Karimunjawa Island Archipelago in the Java Sea. Irish Journal of Medical Science. 1961; 331–48. doi:10.1007/BF02953363

171.

GRIN EI. Endemic Treponematoses in the Sudan. A Report on a Survey. Bulletin of the World Health Organization. 1961;24: 229–38.

172.

GUIMARÄES FN. The Present State of the Yaws Eradication Campaign in Brazil. Boletín de la Oficina Sanitaria Panamericana. 1961;50: 241–5.

173.

JELLIFFE DB, BENNETT FJ, WHITE RHR, CULLINAN TR, JELLIFFE EFP. The Children of the Lugbara. A Study in the Techniques of Paediatric Field Survey in Tropical Africa. Tropical and Geographical Medicine. 1962;14: 33–50.

174.

LA POMMERAY HB, REYES S, DELVA H. Research on Ulcers and their relation to Yaws in Haiti. Boletín de la Oficina Sanitaria Panamericana. 1962;53: 313–16.

175.

ONORI E. The Results of a Mass Campaign against Yaws in the Yolta Region of Ghana. Ghana Medical Journal. 1962;1: 36–45.

176.

Organización Panamericana de la Salud. Reported cases of notifiable diseases in the Americas 1959-1960 [Internet]. Washington, D.C: Organizacion Panamericana de la Salud; 1962. Report No.: Publicación Científica 58. Available: http://apps.who.int/iris/handle/10665/170220

177.

Pan American Health Organization. Summary of four-year reports on health conditions in the Americas 1957-1960 [Internet]. Washington, D.C: Organizacion Panamericana de la Salud; 1962. Available: http://iris.paho.org/xmlui/handle/123456789/1347

178.

PEREIRA EDC. Factors in the Recession of Yaws in Ceylon. Brit J Venereal Dis. 1962;38: 94–8.

179.

PEREIRA EDC. Yaws Control in Ceylon. Brit J Venereal Dis. 1962;38: 90–93.

180.

LEES RE, DE BRUIN AM. Review of yaws in St. Lucia five years after an eradication campaign. West Indian Med J. 1963;12: 98–102.

181.

Organización Panamericana de la Salud. Reported cases of notifiable diseases in the Americas 1961 [Internet]. Washington, D.C: Organizacion Panamericana de la Salud; 1963. Report No.: Publicación Científica 86. Available: http://apps.who.int/iris/handle/10665/167975

182.

ROSEI L. Control of Endemic Yaws in the Brong Ahafo Region of Ghana. West African Medical Journal. 1963; 24–40.

183.

Wang D. Final Report May-December 1962 for Yaws Control project in Kingdom of Tonga. World Health Organization; 1963.

184.

World Health Organization. Second report on the world health situation, 1957-1960 [Internet]. Geneva: World Health Organization; 1963. Report No.: 122. Available: http://apps.who.int/iris/handle/10665/85752

185.

Executive Board, 34. Review of the Organization’s programme in endemic treponematoses and venereal infections: report by the Director-General [Internet]. World Health Organization; 1964. Available: http://apps.who.int/iris/bitstream/10665/137186/1/EB34_11_eng.pdf

186.

GILLES HM. Akufo. An Environmental Study of a Nigerian Village Community. [Internet]. Ibadan: Ibadan University Press, University of Ibadan, Nigeria.; 1964. Available: https://www.cabdirect.org/cabdirect/abstract/19652900796

187.

Organización Panamericana de la Salud. Reported cases of notifiable diseases in the Americas 1962 [Internet]. Washington, D.C: Organizacion Panamericana de la Salud; 1964. Report No.: Publicación Científica 102. Available: http://apps.who.int/iris/handle/10665/169730

188.

Pan American Health Organization. Health conditions in the Americas 1961-1962 [Internet]. Washington, D.C: Organizacion Panamericana de la Salud; 1964. Available: http://iris.paho.org/xmlui/handle/123456789/1145

189.

PAN AMERICAN SANITARY BUREAU., editor. Notifiable Diseases in the Americas, 1961. WHO Chronicle. 1964;18: 155–63.

190.

SAXENA VB, PRASAD BG. An Epidemiological Study of Yaws in Madhya Pradesh: Effectiveness of Preventive Measures. Indian Journal of Medical Research. 1964;52: 1012–33.

191.

SAXENA VB, PRASAD BG. An Epidemiological Study of Yaws in Madhya Pradesh: Its Prevalence and Status. Indian Journal of Medical Research. 1964;52: 999–1011.

192.

World Health Assembly. Supplement to the second report on the world health situation [Internet]. Geneva: World Health Organization; 1964. Report No.: A17/P&B/7. Available: http://apps.who.int/iris/handle/10665/136495

193.

ASHCROFT MT, URQUHART AE, GENTLE GHK. Treponemal Serological Tests in Jamaican School Children. Transactions of the Royal Society of Tropical Medicine and Hygiene. 1965;59: 649–56. doi:10.1016/0035-9203(65)90094-5

194.

AYYANGAR MCR. Some Observations on the Epidemiology and Manifestations of Yaws. Journal of the Indian Medical Association. 1965;44: 190–96.

195.

BRUCE-CHWATT LJ. INTERNATIONAL WORK IN ENDEMIC TREPONEMATOSES AND VENEREAL INFECTIONS, 1948-1963. WHO Chron. 1965;19: 7–18.

196.

CRUZ AH, JUSTINIANO GSD Jr, CAMENA TC. A Critical Evaluation of Yaws in the Philippines. Journal of the Philippine Islands Medical Association. 1965;41: 219–59.

197.

GENTLE GHK. Yaws Survey Jamaica, 1963. Brit J Venereal Dis. 1965;41: 155–62.

198.

GOURLAY RJ, MARSH M. An Outbreak of Yaws in a Suburban Community in Jamaica. American Journal of Tropical Medicine and Hygiene. 1965;14: 777–9.

199.

Organización Panamericana de la Salud. Reported cases of notifiable diseases in the Americas 1963 [Internet]. Washington, D.C: Organizacion Panamericana de la Salud; 1965. Report No.: Publicación Científica 114. Available: http://apps.who.int/iris/handle/10665/169933

200.

GART JJ, DE VRIES JL. The Mathematical Analysis of Concurrent Epidemics of Yaws and Chickenpox. Journal of Hygiene, Cambridge. 1966;64: 431–9.

201.

Organización Panamericana de la Salud. Reported cases of notifiable diseases in the Americas 1964 [Internet]. Washington, D.C: Organizacion Panamericana de la Salud; 1966. Report No.: Publicación Científica 135. Available: http://apps.who.int/iris/handle/10665/170031

202.

Pan American Health Organization. Health conditions in the Americas 1961-1964 [Internet]. Washington, D.C: Organizacion Panamericana de la Salud; 1966. Available: http://iris.paho.org/xmlui/handle/123456789/28381

203.

LEES REM, GENTLE GHK. Yaws control: report of a mass juvenile sweep in St. Lucia. W, Indian Med J. 1967;16: 228–32.

204.

NIGERIA NMOH. Rural health report 1965. Including annual report of the Sleeping Sickness Service for 1965, annual report of the Medical Field Units for 1965. [Internet]. Kaduna: Govt. Printer.; 1967 p. ii+61 pp. Available: https://www.cabdirect.org/cabdirect/abstract/19682901580

205.

NIGERIA NMOH. Rural health report 1966. Including annual report of the Sleeping Sickness Service for 1966, annual report of the Medical Field Units for 1966. [Internet]. Kaduna: Govt. Printer.; 1967 p. iii + 98 pp. Available: https://www.cabdirect.org/cabdirect/abstract/19682903147

206.

Organización Panamericana de la Salud. Reported cases of notifiable diseases in the Americas 1965 [Internet]. Washington, D.C: Organizacion Panamericana de la Salud; 1967. Report No.: Publicación Científica 149. Available: http://apps.who.int/iris/handle/10665/170046

207.

TANEJA BL. Yaws-incidence and epidemiology. (Study in Dudhi Tehsil of District Mirzapur, Uttar Pradesh, India.). Journal of Tropical Medicine and Hygiene. 1967;70: 215–23.

208.

Wasito DR. Assignment Report on Yaws Eradication Programme WHO Project: India. World Health Organization; 1967.

209.

Wong TH. Yaws Control Project, Sierra Leone (1961-1967). World Health Organization; 1967.

210.

World Health Organization. Third report on the world health situation, 1961-1964 [Internet]. Geneva: World Health Organization; 1967. Report No.: 155. Available: http://apps.who.int/iris/handle/10665/85794

211.

GUTHE T, IDSOE O. The rise and fall of the treponematoses. II. Endemic treponematoses of childhood. Brit J Venereal Dis. 1968;44: 35–48.

212.

World Health Assembly. Supplement to the third report on the World Health Situation [Internet]. Geneva: World Health Organization; 1968. Report No.: A21/P&B/3. Available: http://apps.who.int/iris/handle/10665/136495

213.

ASHCROFT MT, BEADNELL HMSG, MILLER GJ, URQUHART AE. VDRL tests in representative communities of Guyanese adults. Brit J Venereal Dis. 1969;45: 140–43.

214.

Organización Panamericana de la Salud. Reported cases of notifiable diseases in the Americas 1966 [Internet]. Washington, D.C: Organizacion Panamericana de la Salud; 1969. Report No.: Publicación Científica 186. Available: http://apps.who.int/iris/handle/10665/170107

215.

TVERSKOY MD. Framboesia in Indonesia. (Clinical manifestations and treatment). Vestnik Dermatologii i Venerologii. 1969;43: 59–67.

216.

GARNER MF, BACKHOUSE JL, TIBBS GJ. Yaws in an isolated Australian aboriginal population. Bulletin of the World Health Organization. 1970;43: 603–6.

217.

GARNER MF, HONABROOK RW. 1968 survey of treponematosis in the Eastern Highlands of New Guinea. Brit J Venereal Dis. 1970;46: 13–17.

218.

Organización Panamericana de la Salud. Reported cases of notifiable diseases in the Americas 1967 [Internet]. Washington, D.C: Organizacion Panamericana de la Salud; 1970. Report No.: Publicación Científica 199. Available: http://apps.who.int/iris/handle/10665/170122

219.

Pan American Health Organization. Health conditions in the Americas 1965-1968 [Internet]. Washington, D.C: Organizacion Panamericana de la Salud; 1970. Available: http://iris.paho.org/xmlui/handle/123456789/1256

220.

SCHALLER KF. Treponematoses in Ethiopia. International Journal of Dermatology. 1970;9: 170–72. doi:10.1111/j.1365-4362.1970.tb05108.x

221.

Organización Panamericana de la Salud. Reported cases of notifiable diseases in the Americas 1968 [Internet]. Washington, D.C: Organizacion Panamericana de la Salud; 1971. Report No.: Publicación Científica 223. Available: http://apps.who.int/iris/handle/10665/170148

222.

SMITH JL, et al. Neuro-ophthalmological study of late yaws and pinta. II. The Caracas Project. Brit J Venereal Dis. 1971;47: 226–51.

223.

World Health Organization. Fourth report on the world health situation, 1965-1968 [Internet]. Geneva: World Health Organization; 1971. Report No.: 192. Available: http://apps.who.int/iris/handle/10665/85832

224.

GARNER MF, BACKHOUSE JL, MOODIE PM, TIBBS GJ. Treponemal infection in the Australian Northern Territory Aborigines. Bulletin of the World Health Organization. 1972;46: 285–93.

225.

GARNER MF, HORNABROOK RW, BACKHOUSE JL. Prevalence of yaws on Kar Kar Island, New Guinea. Brit J Venereal Dis. 1972;48: 350–55.

226.

Guthe T, Ridet J, Vorst F, D’Costa J, Grab B. Methods for the surveillance of endemic treponematoses and sero-immunological investigations of “disappearing” disease. Bull World Health Organ. 1972;46: 1–14.

227.

World Health Assembly. Supplement to the fourth report on the World Health situation [Internet]. Geneva: World Health Organization; 1972. Report No.: A25/16. Available: http://apps.who.int/iris/handle/10665/145465

228.

GARNER MF, HORNABROOK RW. Treponematosis in Papua New Guinea-a review of surveys undertaken between 1964 and 1972. Papua New Guinea Medical Journal. 1973;16: 189–93.

229.

LEES REM. A selective approach to yaws control. Canadian Journal of Public Health. 1973;64: 52–6.

230.

MEDINA R, DIAZ RI, GALLOSO R. The campaign against yaws in Venezuela. The result of three decades of health measures. Dermatologia Venezolana. 1974;13: 1–20.

231.

MORRIS DE, STANDARD KL. Notifiable diseases in the English-speaking Caribbean for the two-year period 1972 and 1973. West Indian Medical Journal. 1974;23: 212–216.

232.

Pan American Health Organization. Health conditions in the Americas 1969-1972 [Internet]. Washington, D.C: Organizacion Panamericana de la Salud; 1974. Available: http://iris.paho.org/xmlui/handle/123456789/28378

233.

World Health Assembly. Fifth report on the world health situation [Internet]. Geneva: World Health Organization; 1974. Report No.: A27/10. Available: http://apps.who.int/iris/handle/10665/146261

234.

PAMPIGLIONE S, RICCIARDI ML. Parasitological survey on Pygmies in Central Africa. II. Bayaka and Badjelli groups (Cameroun). Rivista di Parassitologia. 1975;36: 89–108.

235.

PAMPIGLIONE S, WILKINSON AE. A study of yaws among pygmies in Cameroon and Zaire. Brit J Venereal Dis. 1975;51: 165–169.

236.

WHO. Yaws. Wkly Epidemiol Rec. 1975;50: 145–152.

237.

GRELL G, WATTY E. An analysis of infections and infestations in Dominica, West Indies. West Indian Medical Journal. 1976;25: 166–176.

238.

CIRERA P, PALISSON MJ, PINERD G, JAEGER G. Treponemic serology in a Central African Bi-Aka Pygmy population. Bulletin de la Société de Pathologie Exotique. 1977;70: 32–36.

239.

HÉLÉNON MR. Yaws in Martinique. Bordeaux Medical. 1977;10: 699–701.

240.

HOPKINS DR, FLOREZ D. Comparison of RPR â teardrop â card test, VDRL, and FTA-ABS test results on sera from persons with suspected yaws in Colombia. Brit J Venereal Dis. 1977;53: 218–220.

241.

Hopkins DR, Florez D. Pinta, yaws, and venereal syphilis in Colombia. Int J Epidemiol. 1977;6: 349–355.

242.

Hopkins DR. Yaws in the Americas, 1950-1975. J Infect Dis. 1977;136: 548–554.

243.

Pan American Health Organization. Health conditions in the Americas 1973-1976 [Internet]. Washington, D.C: Organizacion Panamericana de la Salud; 1978. Available: http://iris.paho.org/xmlui/handle/123456789/28377

244.

ADEKOLU-JOHN EO. A communication on health and development in the Kainji Lake area of Nigeria. Acta Tropica. 1979;36: 91–102.

245.

Niemel PL, Brunings EA, Menke HE. Attenuated yaws in Surinam. Br J Vener Dis. 1979;55: 99–101.

246.

Loos M, Dierich MP. Analysis of the anticomplementary activity in sera of three African patients with parasitic and bacteriological infections. Infect Immun. 1980;27: 1–5.

247.

WIDY-WIRSKI R, D’COSTA J, MEHEUS A. Prevalence of yaws in pygmies of the Central African Republic. Annales de la Societe Belge de Medecine Tropicale. 1980;60: 61–67.

248.

World Health Organization. Sixth report on the world health situation: part 2, review by country and area [Internet]. Geneva: World Health Organization; 1980. Available: http://apps.who.int/iris/handle/10665/44199

249.

Osei L. Yaws in rural Accra. J Hyg Epidemiol Microbiol Immunol. 1981;25: 293–300.

250.

WHO. Endemic Treponematoses. Wkly Epidemiol Rec. 1981;56: 241–248.

251.

WHO Regional Office for the Western Pacific. Western Pacific Region data bank on socioeconomic and health indicators, 1980 [Internet]. Manila; 1981. Available: http://apps.who.int/iris/handle/10665/207588

252.

CDC. Yaws and yellow fever project--Ghana. MMWR Morb Mortal Wkly Rep. 1982;31: 149–150.

253.

TOURE IM. Serological and clinical study of yaws in the coastal and central regions of Togo. Medecine d’Afrique Noire. 1982;29: 191–202.

254.

WHO. Surveillance of treponematoses: serological study. Weekly Epidemiological Record. 1982;57: 334–335.

255.

WHO. Yaws and yellow fever surveillance. Weekly Epidemiological Record. 1982;57: 142.

256.

WHO Scientific Group on Treponemal Infections. Les infections tréponémiques : rapport d’un groupe scientifique de l’ OMS [réuni à Genève du 6 au 12 octobre 1980] [Internet]. Genève : Organisation mondiale de la Santé; 1982. Available: http://apps.who.int/iris/bitstream/10665/40907/1/WHO_TRS_674_fre.pdf

257.

WHO Scientific Group on Treponemal Infections. Treponemal infections : report of a WHO scientific group [meeting held in Geneva from 6 to 12 October 1980] [Internet]. Geneva: World Health Organization; 1982 p. 75. Report No.: 674. Available: http://apps.who.int/iris/bitstream/10665/40903/1/WHO_TRS_674.pdf

258.

Agadzi VK, Aboagye-Atta Y, Nelson JW, Perine PL, Hopkins DR. Resurgence of yaws in Ghana. Lancet. 1983; 389–390.

259.

Edorh AA, Dzotsi KA, Deglo AM, Dakey EK. Yaws endemic in Togo. Afrique Médicale. 1983;22: 133–136.

260.

WHO Regional Office for the Western Pacific. Western Pacific Region data bank on socioeconomic and health indicators, 1980 [Internet]. Manila; 1983. Available: http://apps.who.int/iris/handle/10665/207590

261.

Baudon D, Houssou B. Prevalence of yaws in southern Benin in 1982. Annales de la Société Belge de Médecine Tropical. 1984;64: 397–402.

262.

Pan Américan Health Organization, Fogarty International Center. Yaws and endemic treponematoses: Symposium documents. 1984; 317–317.

263.

WHO. SURVEILLANCE OF TREPONEMATOSES. Wkly Epidemiol Rec. 1984;59: 377–384.

264.

WHO Regional Office for the Western Pacific. Western Pacific Region data bank on socioeconomic and health indicators, 1984 [Internet]. Manila; 1984. Available: http://apps.who.int/iris/handle/10665/207591

265.

Abu Ahmed Mohamed H. Endemic treponematoses in the Sudan. Rev Infect Dis. 1985;7 Suppl 2: S239-241.

266.

Agadzi VK, Aboagye-Atta Y, Nelson JW, Hopkins DR, Perine PL. Impact of the control of endemic treponemal diseases in Ghana on other diseases. Rev Infect Dis. 1985;7 Suppl 2: S332-334.

267.

Agadzi VK, Aboagye-Atta Y, Nelson JW, Hopkins DR, Perine PL. Yaws in Ghana. Rev Infect Dis. 1985;7 Suppl 2: S233-236.

268.

Baudon D, Yada A, Roux J. [Extent of endemic yaws in Upper Volta in 1981]. Trop Med Parasitol. 1985;36: 58–60.

269.

Eason RJ, Somerfield SD, Tasman-Jones T, Jones G, Henry A. Resurgent yaws in the Solomon Islands. Australian and New Zealand Journal of Medicine. 1985;15: 727–730.

270.

Eason RJ, Tasman-Jones T. Resurgent yaws and other skin diseases in the Western Province of the Solomon Islands. Papua New Guinea Medical Journal. 1985;28: 247–250.

271.

Heymer A. Sedentarization, acculturation and infectious diseases: socioecological issues for Baka Pygmies. Bulletin de la Societe de Pathologie Exotique et de ses Filiales. 1985;78: 226–238.

272.

Lo EK. Yaws in Malaysia. Rev Infect Dis. 1985;7 Suppl 2: S251-253.

273.

N’Da K. Some epidemiologic aspects of yaws in the Ivory Coast. Rev Infect Dis. 1985;7 Suppl 2: S237-238.

274.

Niemel PL, Sadal S, van der Sluis JJ. Yaws in Suriname. Rev Infect Dis. 1985;7 Suppl 2: S273-275.

275.

Prussia PR, DaSilva PA. Yaws in Barbados. West Indian Medical Journal. 1985;34: 63–65.

276.

Reid MS. Yaws in Papua New Guinea: extent of the problem and status of control programs. Rev Infect Dis. 1985;7 Suppl 2: S254-259.

277.

St John RK. Yaws in the Americas. Rev Infect Dis. 1985;7 Suppl 2: S266-272.

278.

Toure IM. Endemic treponematoses in Togo and other west African states. Rev Infect Dis. 1985;7 Suppl 2: S242-244.

279.

Uribe WR. Yaws in Colombia. Rev Infect Dis. 1985;7 Suppl 2: S276-277.

280.

WHO Regional Office for the Western Pacific. Western Pacific Region data bank on socioeconomic and health indicators, 1985 [Internet]. Manila; 1985. Available: http://apps.who.int/iris/handle/10665/207597

281.

Widy-Wirski R. Surveillance and control of resurgent yaws in the African region. Rev Infect Dis. 1985;7 Suppl 2: S227-232.

282.

Willcox RR. Mass treatment campaigns against the endemic treponematoses. Rev Infect Dis. 1985;7 Suppl 2: S278-283.

283.

Zahra A. Yaws in Southeast Asia: an overview. Rev Infect Dis. 1985;7 Suppl 2: S245-250.

284.

Ziefer A, Lanoie LO, Meyers WM, Vanderpas J, Charon F, Connor DH. Studies on a focus of yaws in Ubangi, Zaire. Trop Med Parasitol. 1985;36: 63–71.

285.

International symposium on yaws and other endemic treponematoses held in Washington DC, 16-18 April 1984continued. 1985. pp. S217–S351. Available: https://www.cabdirect.org/cabdirect/abstract/19862026108

286.

Alemaena O. Yaws situation in the Solomon Islands. Southeast Asian J Trop Med Public Health. 1986;17: 14–18.

287.

Antal G, Burke J, Geizer I, Lukehart SA, editors. Proceedings of inter-regional meeting on yaws and other endemic treponematoses. Cipanas, Indonesia, July 22-24, 1985. Southeast Asian J Trop Med Public Health. 1986;17: 1–96.

288.

Dutta M. Yaws in India. Southeast Asian J Trop Med Public Health. 1986;17: 35–41.

289.

Geizer I. Yaws in the Western Pacific region: an overview. Southeast Asian J Trop Med Public Health. 1986;17: 8–13.

290.

Jayakuru GN. Yaws in Sri Lanka. Southeast Asian J Trop Med Public Health. 1986;17: 42–48.

291.

Larsen SA, D’Costa JF. Laboratory tests and serologic surveillance for yaws and other diseases and conditions. Southeast Asian J Trop Med Public Health. 1986;17: 70–77.

292.

Narain JP, Basu RN, Ray SN, Sharma RS. Extent of yaws problem in India. Journal of Communicable Diseases. 1986;18: 128–131.

293.

Omar MA. Yaws assessment in Somalia. Southeast Asian J Trop Med Public Health. 1986;17: 66–69.

294.

Sanyakorn CK. Situation of control of yaws in the Southeast Asia region: past, present, and future. Southeast Asian J Trop Med Public Health. 1986;17: 3–7.

295.

Sosroamidjojo S, Rai K, Suesen N. Yaws in Indonesia. Southeast Asian J Trop Med Public Health. 1986;17: 19–34.

296.

Talwat E. Papua New Guinea. Yaws problems assessed. Southeast Asian J Trop Med Public Health. 1986;17: 59–65.

297.

Traisupa A. Yaws in Thailand. Southeast Asian J Trop Med Public Health. 1986;17: 49–58.

298.

WHO. Endemic Treponematoses. Wkly Epidemiol Rec. 1986;61: 197–204.

299.

WHO. ENDEMIC TREPONEMATOSES: Outbreak of yaws infections. Wkly Epidemiol Rec. 1986;61: 141–148.

300.

WHO. Yaws and other endemic treponematoses : report of a regional meeting, Brazzaville, 3-6 February 1986 [Internet]. Brazzaville: World Health Organization Regional Office for Africa; 1986 p. 73. Available: http://whqlibdoc.who.int/afro/-1993/AFR_CD_58.pdf

301.

Duncan LE, Alto W. An investigation of yaws on the Trobriand Islands, 1985. Papua New Guinea Medical Journal. 1987;30: 57–61.

302.

WHO Regional Office for the Western Pacific. Western Pacific Region data bank on socioeconomic and health indicators, 1987 [Internet]. Manila; 1987. Available: http://apps.who.int/iris/handle/10665/207594

303.

Martin PM, Gonzalez JP, Martin MH, Georges-Courbot MC, Palisson MJ, Georges AJ. Clinical aspects and usefulness of indirect absorbed immunofluorescence for diagnosis of yaws in Central Africa. J Clin Microbiol. 1988;26: 2432–2433.

304.

Mohamed KN. Imported yaws in Johor, Malaysia. Annals of Tropical Paediatrics. 1988;8: 222–224.

305.

Talhari DS. The Present Situation of Endemic Treponematoses (Yaws and Pinta) in the Region of the Americas. Pan American Health Organization; 1988.

306.

WHO Regional Office for the Western Pacific. Western Pacific Region data bank on socioeconomic and health indicators, 1988 [Internet]. Manila; 1988. Available: http://apps.who.int/iris/handle/10665/207596

307.

De Schryver A, Meheus A. [Review: endemic treponematoses are not always eradicated]. Med Trop (Mars). 1989;49: 237–244.

308.

Gip LS. Yaws revisited. Med J Malaysia. 1989;44: 307–311.

309.

WHO Regional Office for the Western Pacific. Western Pacific Region data bank on socioeconomic and health indicators, 1989 [Internet]. Manila; 1989. Available: http://apps.who.int/iris/handle/10665/207596

310.

Engelkens HJ, Ginai AZ, Judanarso J, Kasim C, van der Stek J, van der Sluis JJ, et al. Radiological and dermatological findings in two patients suffering from early yaws in Indonesia. Genitourin Med. 1990;66: 259–263.

311.

Fegan D, Glennon M, Macbride-Stewart G, Moore T. Yaws in the Solomon Islands. Journal of Tropical Medicine and Hygiene. 1990;93: 52–57.

312.

Martin PM, Cockayne A, Georges AJ, Penn CW. Immune response to Treponema pertenue and Treponema pallidum Nichols in patients  with yaws. Res Microbiol. 1990;141: 181–186.

313.

Mohamed KN. Late yaws and optic atrophy. Annals of Tropical Medicine and Parasitology. 1990;84: 637–639.

314.

WHO Regional Office for the Western Pacific. Western Pacific Region data bank on socioeconomic and health indicators, 1990 [Internet]. Manila; 1990. Available: http://apps.who.int/iris/handle/10665/207598

315.

Engelkens HJ, Vuzevski VD, ten Kate FJ, van der Heul P, van der Sluis JJ, Stolz E. Ultrastructural aspects of infection with Treponema pallidum subspecies pertenue  (Pariaman strain). Genitourin Med. 1991;67: 403–407.

316.

Guderian RH, Guzman JR, Calvopiña M, Cooper P. Studies on a focus of yaws in the Santiago Basin, province of Esmeraldas, Ecuador. Tropical and Geographical Medicine. 1991;43: 142–147.

317.

Harris M, Nako D, Hopkins T (et al.). Yaws infection in Tanna, Vanuatu 1989. Southeast Asian Journal of Tropical Medicine and Public Health. 1991;22: 113–119.

318.

Noordhoek GT, Engelkens HJ, Judanarso J, van der Stek J, Aelbers GN, van der Sluis JJ, et al. Yaws in West Sumatra, Indonesia: clinical manifestations, serological findings and characterisation of new Treponema isolates by DNA probes. Eur J Clin Microbiol Infect Dis. 1991;10: 12–19.

319.

WHO Regional Office for the Western Pacific. Western Pacific Region data bank on socioeconomic and health indicators, 1991 [Internet]. Manila; 1991. Available: http://apps.who.int/iris/handle/10665/207602

320.

Engelkens HJ, Ginai AZ, Judanarso J, Stolz E. Case report 724. Yaws. Skeletal Radiol. 1992;21: 194–197.

321.

Engelkens HJH, Stolz E. A small yaws survey on the island of Sumatra, Indonesia. Acta Leidensia. 1992;60: 19–29.

322.

Gershman KA, Rolfs RT, Larsen SA, Zaidi A, Palafox NA. Seroepidemiological characterization of a syphilis epidemic in the Republic of the Marshall Islands, formerly a yaws endemic area. Int J Epidemiol. 1992;21: 599–606.

323.

Hervé V, Kassa Kelembho E, Normand P, Georges A, Mathiot C, Martin P. Resurgence of yaws in the Central African Republic. Role of the Pygmy population as a reservoir of the pathogen. Bulletin de la Société de Pathologie Exotique. 1992;85: 342–346.

324.

Meheus A, Antal GM. The endemic treponematoses: not yet eradicated. World Health Stat Q. 1992;45: 228–237.

325.

WHO Regional Office for the Western Pacific. Western Pacific Region data bank on socioeconomic and health indicators, 1992 [Internet]. Manila; 1992. Available: http://apps.who.int/iris/handle/10665/207603

326.

Engelkens HJ, ten Kate FJ, Judanarso J, Vuzevski VD, van Lier JB, Godschalk JC, et al. The localisation of treponemes and characterisation of the inflammatory infiltrate in skin biopsies from patients with primary or secondary syphilis, or  early infectious yaws. Genitourin Med. 1993;69: 102–107.

327.

Richens J. Whatever happened to yaws eradication? Africa Health. 1993;15: 14–17.

328.

Edorh AA, Siamevi EK, Adanlete FA, Aflagah EK, Kassankogno Y, Amouzou AB, et al. [Resurgence of endemic yaws in Togo. Cause and eradication approach]. Bull Soc Pathol Exot. 1994;87: 17–18.

329.

Pan American Health Organization. Health conditions in the Americas, 1994 edition, v.2 [Internet]. Washington, D.C: Organizacion Panamericana de la Salud; 1994. Available: http://iris.paho.org/xmlui/handle/123456789/28370

330.

Sehgal VN, Jain S, Bhattacharya SN, Thappa DM. Yaws control/eradication. Int J Dermatol. 1994;33: 16–20.

331.

Tharmaphornpilas P, Srivanichakorn S, Phraesrisakul N. Recurrence of yaws outbreak in Thailand, 1990. Southeast Asian J Trop Med Public Health. 1994;25: 152–156.

332.

WHO Regional Office for the Western Pacific. Western Pacific Region data bank on socioeconomic and health indicators, 1994 [Internet]. Manila; 1994. Available: http://apps.who.int/iris/handle/10665/207606

333.

Anselmi M, Araujo E, Narváez A, Cooper PJ, Guderian RH. Yaws in Ecuador: impact of control measures on the disease in the Province of Esmeraldas. Genitourinary Medicine. 1995;71: 343–346.

334.

Backhouse JL, Hudson BJ. Evaluation of immunoglobulin G enzyme immunoassay for serodiagnosis of yaws. Journal of Clinical Microbiology. 1995;33: 1875–1878.

335.

Guderian RH, Anselmi M, Calvopiña M, Cooper PJ, Mancero T. El pian en la provincia de Esmeraldas, Ecuador. Biomedica. 1995;15: 137–43.

336.

WHO Regional Office for the Western Pacific. Western Pacific Region data bank on socioeconomic and health indicators, 1995 [Internet]. Manila; 1995. Available: http://apps.who.int/iris/handle/10665/207607

337.

World Health Organization. Division of Emerging, Viral and Bacterial Diseases Surveillance and Control. Informal consultation on endemic treponematoses : report of an informal consultation, Geneva, Switzerland, 6-7 July 1995. Geneva: World Health Organization; 1995 p. 10.

338.

WHO Regional Office for the Western Pacific. Country health information profiles : 1997 revision [Internet]. Manila: WHO Regional Office for the Western Pacific; 1997. Available: http://apps.who.int/iris/handle/10665/207702

339.

Yedomon HG, Ango-Padonou F do. Treponemic serology: interpretation in 326 cases. Médecine d’Afrique Noire. 1997;44: 229–232.

340.

Backhouse JL, Hudson BJ, Hamilton PA, Nesteroff SI. Failure of penicillin treatment of yaws on Karkar Island, Papua New Guinea. American Journal of Tropical Medicine and Hygiene. 1998;59: 388–392.

341.

Akogun OB. Yaws and syphilis in the Garkida area of Nigeria. Zentralblatt für Bakteriologie. 1999;289: 101–107.

342.

Engelkens HJH, Vuzevski VD, Stolz E. Nonvenereal treponematoses in tropical countries. Clinics in Dermatology. 1999;17: 143–152. doi:10.1016/S0738-081X(99)00007-3

343.

WHO Regional Office for the Western Pacific. Country health information profiles : 1999 revision [Internet]. Manila: WHO Regional Office for the Western Pacific; 1999. Available: http://apps.who.int/iris/handle/10665/207586

344.

Walker SL, Hay RJ. Yaws-a review of the last 50 years. Int J Dermatol. 2000;39: 258–260.

345.

Restrepo M, Restrepo BN, Orozco B, Padilla JC, Cuervo CM, Alvarez G. Seroepidemiological survey of yaws on the Pacific Coast of Colombia. Biomédica. 2001;21: 155–161.

346.

Manning LA, Ogle GD. Yaws in the periurban settlements of Port Moresby, Papua New Guinea. Papua New Guinea Medical Journal. 2002;45: 206–212.

347.

Anselmi M, Moreira JM, Caicedo C, Guderian R, Tognoni G. Community participation eliminates yaws in Ecuador. Tropical Medicine and International Health. 2003;8: 634–638. doi:10.1046/j.1365-3156.2003.01073.x

348.

Noray G de, Capuano C, Abel M. Campaign to eradicate yaws on Santo Island, Vanuatu in 2001. Médecine Tropicale. 2003;63: 159–162.

349.

Scolnik D, Aronson L, Lovinsky R, Toledano K, Glazier R, Eisenstadt J, et al. Efficacy of a targeted, oral penicillin-based yaws control program among children living in rural South America. Clinical Infectious Diseases. 2003;36: 1232–1238. doi:10.1086/374338

350.

Bora D, Dhariwal AC, Shiv Lal. Yaws and its eradication in India - a brief review. Journal of Communicable Diseases. 2005;37: 1–11.

351.

WHO Regional Office for the Western Pacific. Western Pacific country health information profiles : 2006 revision [Internet]. Manila: WHO Regional Office for the Western Pacific; 2006. Available: http://apps.who.int/iris/handle/10665/208188

352.

Chandrakant Lahariya, Pradhan SK. Can Southeast Asia eradicate yaws by 2010? Some lessons from the Yaws Eradication Programme of India. National Medical Journal of India. 2007;20: 81–86.

353.

Konan YE, M’Bea KJ, Coulibaly A, Tetchi EO, Kpebo DOD, Ake O, et al. [A description of the yaws infection and prevention conditions in the health district of Adzope]. Sante Publique. 2007;19: 111–118.

354.

Touré B, Koffi NM, Assi KP, Ake O, Konan DJP. Yaws in Côte d’Ivoire: health problem forgotten and neglected. Bulletin de la Société de Pathologie Exotique. 2007;100: 130–132.

355.

WHO Regional Office for the Western Pacific. Western Pacific country health information profiles : 2007 revision [Internet]. Manila: WHO Regional Office for the Western Pacific; 2007. Available: http://apps.who.int/iris/handle/10665/208183

356.

Asiedu K, Amouzou B, Dhariwal A, Karam M, Lobo D, Patnaik S, et al. Yaws eradication: past efforts and future perspectives. Bull World Health Organ. 2008;86: 499–499A.

357.

Pepin J, Labbe A-C. Noble goals, unforeseen consequences: control of tropical diseases in colonial Central Africa and the iatrogenic transmission of blood-borne viruses. Trop Med Int Health. 2008;13: 744–753. doi:10.1111/j.1365-3156.2008.02060.x

358.

WHO Regional Office for the Western Pacific. Western Pacific country health information profiles : 2008 revision [Internet]. Manila: WHO Regional Office for the Western Pacific; 2008. Available: http://apps.who.int/iris/handle/10665/208187

359.

World Health Organization, editor. Elimination of yaws in India. Weekly Epidemiological Record. 2008;83: 125–132.

360.

World Health Organization, editor. Meeting of the International Task Force for Disease Eradication - 11 October 2007. Weekly Epidemiological Record. 2008;83: 77–81.

361.

Gerstl S, Kiwila G, Dhorda M, Lonlas S, Myatt M, Ilunga BK, et al. Prevalence study of yaws in the Democratic Republic of Congo using the lot quality assurance sampling method. PLoS One. 2009;4: e6338. doi:10.1371/journal.pone.0006338

362.

WHO Regional Office for the Western Pacific. Western Pacific country health information profiles : 2009 revision [Internet]. Manila: WHO Regional Office for the Western Pacific; 2009. Available: http://apps.who.int/iris/handle/10665/208186

363.

Fegan D, Glennon MJ, Thami Y, Pakoa G. Resurgence of yaws in Tanna, Vanuatu: time for a new approach? Tropical Doctor. 2010;40: 68–69. doi:10.1258/td.2009.090249

364.

Santos MML dos, Amaral S, Harmen SP, Joseph HM, Fernandes JL, Counahan ML. The prevalence of common skin infections in four districts in Timor-Leste: a cross sectional survey. BMC Infectious Diseases. 2010;10: (10 March 2010).

365.

WHO Regional Office for the Western Pacific. Western Pacific country health information profiles : 2010 revision [Internet]. Manila: WHO Regional Office for the Western Pacific; 2010. Available: http://apps.who.int/iris/handle/10665/207482

366.

World Health Organization, editor. Communicable disease epidemiological profile: Côte d’Ivoire. [Internet]. Geneva: World Health Organization; 2010. Available: http://whqlibdoc.who.int/hq/2010/WHO_HSE_GAR_DCE_2010.3_eng.pdf

367.

Capuano C, Ozaki M. Yaws in the Western Pacific Region: a review of the literature. Journal of Tropical Medicine. 2011;2011: Article ID 642832. doi:10.1155/2011/642832

368.

Guerrier G, Marcon S, Garnotel L, Deltour R, Schinas S, Mathelin JP, et al. Yaws in Polynesia’s Wallis and Futuna Islands: a seroprevalence survey. N Z Med J. 2011;124: 29–31.

369.

Manirakiza A, Boas SV, Beyam N, Zadanga G, Konamna FX, Njuimo SP, et al. Clinical outcome of skin yaws lesions after treatment with benzathinebenzylpenicillin in a pygmy population in Lobaye, Central African Republic. BMC Res Notes. 2011;4: 543. doi:10.1186/1756-0500-4-543

370.

Mitja O, Hays R, Ipai A, Gubaila D, Lelngei F, Kirara M, et al. Outcome predictors in treatment of yaws. Emerg Infect Dis. 2011;17: 1083–1085. doi:10.3201/eid/1706.101575

371.

Mitja O, Hays R, Ipai A, Wau B, Bassat Q. Osteoperiostitis in early yaws: case series and literature review. Clin Infect Dis. 2011;52: 771–774. doi:10.1093/cid/ciq246

372.

Mitja O, Hays R, Lelngei F, Laban N, Ipai A, Pakarui S, et al. Challenges in recognition and diagnosis of yaws in children in Papua New Guinea. Am J Trop Med Hyg. 2011;85: 113–116. doi:10.4269/ajtmh.2011.11-0062

373.

Pillay A, Chen C-Y, Reynolds MG, Mombouli JV, Castro AC, Louvouezo D, et al. Laboratory-confirmed case of yaws in a 10-year-old boy from the Republic of the Congo. J Clin Microbiol. 2011;49: 4013–4015. doi:10.1128/JCM.01121-11

374.

WHO Regional Office for the Western Pacific. Western Pacific country health information profiles : 2011 revision [Internet]. Manila: WHO Regional Office for the Western Pacific; 2011. Available: http://apps.who.int/iris/handle/10665/208132

375.

Lim KG. The Eradication of Yaws - and its unsung hero. Med J Malaysia. 2012;67: 557–559.

376.

Mitjà O, Hays R, Ipai A, Penias M, Paru R, Fagaho D, et al. Single-dose azithromycin versus benzathine benzylpenicillin for treatment of yaws in children in Papua New Guinea: an open-label, non-inferiority, randomised trial. Lancet (British edition). 2012;379: 342–347. doi:10.1016/S0140-6736(11)61624-3

377.

Muniz ES. [“Is a shot alone enough?”: concepts of health, hygiene, and nutrition and the Program to Eradicate Yaws in Brazil, 1956-1961]. Hist Cienc Saude Manguinhos. 2012;19: 197–216.

378.

WHO. Regional Office for South-East Asia. Regional strategic plan for elimination of yaws from South-East Asia Region 2012-2020 [Internet]. 2012. Available: http://apps.who.int/iris/bitstream/10665/205830/1/B4948.pdf

379.

World Health Organization, editor. Eradication of yaws - the Morges Strategy. Weekly Epidemiological Record. 2012;87: 189–194.

380.

World Health Organization. Summary report of a consultation on the eradication of yaws, 5-7 March 2012, Morges, Switzerland [Internet]. World Health Organization; 2012. Available: http://apps.who.int/iris/handle/10665/75528

381.

Bratschi MW, Bolz M, Minyem JC, Grize L, Wantong FG, Kerber S, et al. Geographic distribution, age pattern and sites of lesions in a cohort of buruli ulcer patients from the Mapé basin of Cameroon. PLoS Neglected Tropical Diseases. 2013;7: e2252. doi:10.1371/journal.pntd.0002252

382.

Coldiron M, Obvala D, Mouniaman-Nara I, Pena J, Blondel C, Porten K. [The prevalence of yaws among the Aka in the Congo]. Med Sante Trop. 2013;23: 231–232. doi:10.1684/mst.2013.0220

383.

Kline K, McCarthy JS, Pearson M, Loukas A, Hotez PJ. Neglected tropical diseases of Oceania: review of their prevalence, distribution, and opportunities for control. PLoS Neglected Tropical Diseases. 2013;7: e1755. doi:10.1371/journal.pntd.0001755

384.

Konan DJP, Aka J, Yao KJ, Kouassi-Gohou V, Yao KE, Faye-Kette H. [Update on a neglected tropical disease from the routine health information system in Cote d’Ivoire: Yaws, 2001 to 2011]. Med Sante Trop. 2013;23: 433–438. doi:10.1684/mst.2013.0252

385.

Mitjà O, Asiedu K, Mabey D. Yaws. Lancet. 2013; doi:10.1016/S0140-6736(12)62130-8

386.

WHO. Meeting of the International Task Force for Disease Eradication – November 2012. Wkly Epidemiol Rec. 2013;88: 73–80.

387.

Agana-Nsiire P, Kaitoo E, Agongo EEA, Bonsu G, Kyei-Faried S, Amponsa-Achiano K, et al. Yaws Prevalence, Lessons from the Field and the Way Forward towards Yaws Eradication in Ghana. Int Sch Res Notices. 2014;2014: 910937. doi:10.1155/2014/910937

388.

Asiedu K, Fitzpatrick C, Jannin J. Eradication of yaws: historical efforts and achieving WHO’s 2020 target. PLoS Neglected Tropical Diseases. 2014;8: e3016. doi:10.1371/journal.pntd.0003016

389.

Ayove T, Houniei W, Wangnapi R, Bieb SV, Kazadi W, Luke LN, et al. Sensitivity and specificity of a rapid point-of-care test for active yaws: a comparative study. Lancet Global Health. 2014;2: e415–e421. doi:10.1016/S2214-109X(14)70231-1

390.

Jain SK, Thomas TG, Bora D, Venkatesh S. Eradicating yaws from India: a summary. Journal of Communicable Diseases. 2014;46: 1–9.

391.

Kazadi WM, Asiedu KB, Agana N, Mitja O. Epidemiology of yaws: an update. Clin Epidemiol. 2014;6: 119–128. doi:10.2147/CLEP.S44553

392.

Marks M, Solomon AW, Mabey DC. Endemic treponemal diseases. Trans R Soc Trop Med Hyg. 2014;108: 601–607. doi:10.1093/trstmh/tru128

393.

Mitjà O, Lukehart SA, Pokowas G, Moses P, Kapa A, Godornes C, et al. Haemophilus ducreyi as a cause of skin ulcers in children from a yaws-endemic area of Papua New Guinea: a prospective cohort study. Lancet Global Health. 2014;2: e235–e241. doi:10.1016/S2214-109X(14)70019-1

394.

Chi KH, Danavall D, Taleo F, Pillay A, Ye T, Nachamkin E, et al. Molecular differentiation of Treponema pallidum subspecies in skin ulceration clinically suspected as yaws in Vanuatu using real-time multiplex PCR and serological methods. American Journal of Tropical Medicine and Hygiene. 2015;92: 134–138. doi:10.4269/ajtmh.14-0459

395.

Ghinai R, El-Duah P, Chi KH, Pillay A, Solomon AW, Bailey RL, et al. A cross-sectional study of “yaws” in districts of Ghana which have previously undertaken azithromycin mass drug administration for trachoma control. PLoS Neglected Tropical Diseases. 2015;9: e0003496. doi:10.1371/journal.pntd.0003496

396.

Marks M, Katz S, Chi K-H, Vahi V, Sun Y, Mabey DC, et al. Failure of PCR to Detect Treponema pallidum ssp. pertenue DNA in Blood in Latent  Yaws. PLoS Negl Trop Dis. 2015;9: e0003905. doi:10.1371/journal.pntd.0003905

397.

Marks M, Mitja O, Solomon AW, Asiedu KB, Mabey DC. Yaws. Br Med Bull. 2015;113: 91–100. doi:10.1093/bmb/ldu037

398.

Marks M, Vahi V, Sokana O, Chi K-H, Puiahi E, Kilua G, et al. Impact of Community Mass Treatment with Azithromycin for Trachoma Elimination on  the Prevalence of Yaws. PLoS Negl Trop Dis. 2015;9: e0003988. doi:10.1371/journal.pntd.0003988

399.

Marks M, Vahi V, Sokana O, Puiahi E, Pavluck A, Zhang Z, et al. Mapping the epidemiology of yaws in the Solomon Islands: a cluster randomized survey. Am J Trop Med Hyg. 2015;92: 129–133. doi:10.4269/ajtmh.14-0438

400.

Mitja O, Houinei W, Moses P, Kapa A, Paru R, Hays R, et al. Mass treatment with single-dose azithromycin for yaws. N Engl J Med. 2015;372: 703–710. doi:10.1056/NEJMoa1408586

401.

Mitjà O, Marks M, Konan DJP, Ayelo G, Gonzalez-Beiras C, Boua B, et al. Global epidemiology of yaws: a systematic review. Lancet Glob Health. 2015;3: e324-331. doi:10.1016/S2214-109X(15)00011-X

402.

Narain JP, Jain SK, Bora D, Venkatesh S. Eradicating successfully yaws from India: The strategy & global lessons. Indian J Med Res. 2015;141: 608–613.

403.

Solomon AW, Marks M, Martin DL, Mikhailov A, Flueckiger RM, Mitjà O, et al. Trachoma and yaws: common ground? PLoS Neglected Tropical Diseases. 2015;9: e0004071. doi:10.1371/journal.pntd.0004071

404.

WHO. Eradication of yaws in India. Wkly Epidemiol Rec. 2015;90: 161–168.

405.

Marks M, Sokana O, Nachamkin E, Puiahi E, Kilua G, Pillay A, et al. Prevalence of Active and Latent Yaws in the Solomon Islands 18 Months after Azithromycin Mass Drug Administration for Trachoma. PLoS Negl Trop Dis. 2016;10: e0004927. doi:10.1371/journal.pntd.0004927

406.

Sarkodie F, Owusu-Dabo E, Hassall O, Bates I, Bygbjerg IC, Ullum H. Recall of symptoms and treatment of syphilis and yaws by healthy blood donors screening positive for syphilis in Kumasi, Ghana. Int J Infect Dis. 2016;50: 72–74. doi:10.1016/j.ijid.2016.08.006
