# Supplementary material for: Prioritizing surveillance activities for certification of yaws eradication based on a review and model of historical case reporting
Source: PLoS Negl Trop Dis. 2018 Dec 4;12(12):e0006953. doi: 10.1371/journal.pntd.0006953 (PMC6294396; doi:10.1371/journal.pntd.0006953)
Supplement: S2 Table — (DOCX) [file pntd.0006953.s003.docx]

| **Country or area** | **ISO3 code** | **Category** | **Year of last report** | **Urban population share** | **Best estimate** | **Low estimate** | **High estimate** |
| --- | --- | --- | --- | --- | --- | --- | --- |
| Wallis and Futuna Islands | WLF | A.2 | 2010 | 0.00 | 0.06 | 0.02 | 0.14 |
| Malawi | MWI | B.1 | 1950 | 0.16 | 0.07 | 0.03 | 0.13 |
| Zimbabwe | ZWE | B.1 | 1950 | 0.40 | 0.1 | 0.05 | 0.18 |
| South Sudan | SSD | B.1 | 1962 | 0.19 | 0.13 | 0.06 | 0.24 |
| Trinidad and Tobago | TTO | B.1 | 1979 | 0.14 | 0.16 | 0.1 | 0.24 |
| United Republic of Tanzania | TZA | B.1 | 1978 | 0.28 | 0.16 | 0.1 | 0.22 |
| Samoa | WSM | B.1 | 1966 | 0.19 | 0.17 | 0.12 | 0.24 |
| Sri Lanka | LKA | B.1 | 1983 | 0.15 | 0.17 | 0.12 | 0.23 |
| Saint Lucia | LCA | B.1 | 1979 | 0.15 | 0.19 | 0.14 | 0.26 |
| Uganda | UGA | B.1 | 1984 | 0.17 | 0.19 | 0.13 | 0.26 |
| Burundi | BDI | B.1 | 1988 | 0.12 | 0.2 | 0.14 | 0.28 |
| Rwanda | RWA | B.1 | 1989 | 0.20 | 0.2 | 0.15 | 0.27 |
| Sierra Leone | SLE | B.1 | 1981 | 0.40 | 0.22 | 0.16 | 0.3 |
| Zambia | ZMB | B.1 | 1985 | 0.40 | 0.22 | 0.14 | 0.31 |
| Kenya | KEN | B.1 | 1983 | 0.25 | 0.23 | 0.16 | 0.32 |
| Montserrat | MSR | B.1 | 1966 | 0.17 | 0.23 | 0.14 | 0.34 |
| Mozambique | MOZ | B.1 | 1984 | 0.32 | 0.24 | 0.14 | 0.36 |
| China | CHN | B.1 | 1957 | 0.54 | 0.25 | 0.17 | 0.36 |
| Madagascar | MDG | B.1 | 1981 | 0.34 | 0.25 | 0.18 | 0.32 |
| Micronesia (Federated States of) | FSM | B.1 | 1946 | 0.23 | 0.25 | 0.13 | 0.43 |
| Guatemala | GTM | B.1 | 1975 | 0.51 | 0.27 | 0.2 | 0.36 |
| Mauritania | MRT | B.1 | 1950 | 0.42 | 0.27 | 0.1 | 0.52 |
| Saint Kitts and Nevis | KNA | B.1 | 1967 | 0.32 | 0.27 | 0.18 | 0.37 |
| Tonga | TON | B.1 | 1976 | 0.24 | 0.28 | 0.19 | 0.38 |
| Antigua and Barbuda | ATG | B.1 | 1977 | 0.30 | 0.29 | 0.21 | 0.39 |
| Costa Rica | CRI | B.1 | 1957 | 0.66 | 0.29 | 0.18 | 0.42 |
| Equatorial Guinea | GNQ | B.1 | 1984 | 0.40 | 0.29 | 0.16 | 0.46 |
| Paraguay | PRY | B.1 | 1963 | 0.64 | 0.29 | 0.19 | 0.4 |
| Guinea | GIN | B.1 | 1988 | 0.37 | 0.3 | 0.22 | 0.4 |
| Burkina Faso | BFA | B.1 | 1988 | 0.29 | 0.31 | 0.22 | 0.41 |
| Chad | TCD | B.1 | 1986 | 0.22 | 0.31 | 0.13 | 0.53 |
| Niger | NER | B.1 | 1988 | 0.19 | 0.31 | 0.23 | 0.41 |
| British Virgin Islands | VGB | B.1 | 1954 | 0.42 | 0.32 | 0.19 | 0.49 |
| Ethiopia | ETH | B.1 | 1988 | 0.18 | 0.32 | 0.25 | 0.39 |
| Jamaica | JAM | B.1 | 1972 | 0.52 | 0.32 | 0.19 | 0.46 |
| Barbados | BRB | B.1 | 1985 | 0.46 | 0.33 | 0.23 | 0.44 |
| Cambodia | KHM | B.1 | 1991 | 0.21 | 0.33 | 0.23 | 0.45 |
| Somalia | SOM | B.1 | 1950 | 0.39 | 0.33 | 0.13 | 0.59 |
| Bolivia (Plurinational State of) | BOL | B.1 | 1966 | 0.68 | 0.34 | 0.23 | 0.47 |
| Dominican Republic | DOM | B.1 | 1972 | 0.71 | 0.34 | 0.23 | 0.47 |
| Bahamas | BHS | B.1 | 1954 | 0.85 | 0.35 | 0.17 | 0.57 |
| Djibouti | DJI | B.1 | 1950 | 0.77 | 0.35 | 0.12 | 0.65 |
| Senegal | SEN | B.1 | 1988 | 0.43 | 0.35 | 0.26 | 0.46 |
| Gambia | GMB | B.1 | 1983 | 0.59 | 0.36 | 0.25 | 0.5 |
| Viet Nam | VNM | B.1 | 1990 | 0.33 | 0.36 | 0.27 | 0.46 |
| Comoros | COM | B.1 | 1960 | 0.28 | 0.37 | 0.15 | 0.65 |
| Cuba | CUB | B.1 | 1960 | 0.75 | 0.37 | 0.24 | 0.51 |
| Grenada | GRD | B.1 | 1972 | 0.40 | 0.37 | 0.25 | 0.5 |
| Haiti | HTI | B.1 | 1993 | 0.57 | 0.38 | 0.27 | 0.5 |
| Guyana | GUY | B.1 | 2001 | 0.29 | 0.39 | 0.31 | 0.47 |
| Liberia | LBR | B.1 | 1984 | 0.49 | 0.39 | 0.25 | 0.53 |
| Angola | AGO | B.1 | 1988 | 0.61 | 0.42 | 0.29 | 0.56 |
| Guinea-Bissau | GNB | B.1 | 1987 | 0.46 | 0.42 | 0.32 | 0.51 |
| New Caledonia | NCL | B.1 | 1960 | 0.61 | 0.43 | 0.27 | 0.61 |
| Saint Vincent and the Grenadines | VCT | B.1 | 1979 | 0.50 | 0.43 | 0.31 | 0.56 |
| Thailand | THA | B.1 | 2000 | 0.35 | 0.43 | 0.36 | 0.5 |
| India | IND | A.1 | 2003 | 0.32 | 0.46 | 0.39 | 0.52 |
| Lao People's Democratic Republic | LAO | B.1 | 1997 | 0.38 | 0.46 | 0.38 | 0.54 |
| Mali | MLI | B.1 | 1986 | 0.37 | 0.46 | 0.32 | 0.61 |
| Kiribati | KIR | B.1 | 1983 | 0.44 | 0.47 | 0.34 | 0.61 |
| Panama | PAN | B.1 | 1977 | 0.77 | 0.47 | 0.33 | 0.62 |
| Peru | PER | B.1 | 1979 | 0.78 | 0.49 | 0.35 | 0.64 |
| Suriname | SUR | B.1 | 1983 | 0.71 | 0.52 | 0.35 | 0.7 |
| Niue | NIU | B.1 | 1983 | 0.50 | 0.53 | 0.37 | 0.68 |
| Brazil | BRA | B.1 | 1976 | 0.85 | 0.54 | 0.38 | 0.7 |
| French Guiana | GUF | B.1 | 1955 | 0.77 | 0.54 | 0.33 | 0.74 |
| Fiji | FJI | B.1 | 1995 | 0.53 | 0.55 | 0.45 | 0.66 |
| Malaysia | MYS | B.1 | 1990 | 0.75 | 0.57 | 0.44 | 0.69 |
| Tuvalu | TUV | B.1 | 1990 | 0.50 | 0.57 | 0.42 | 0.7 |
| Nigeria | NGA | B.1 | 1996 | 0.51 | 0.59 | 0.5 | 0.68 |
| Togo | TGO | A.2 | 2015 | 0.39 | 0.6 | 0.52 | 0.68 |
| Papua New Guinea | PNG | A.2 | 2015 | 0.13 | 0.61 | 0.51 | 0.7 |
| Australia | AUS | B.1 | 1970 | 0.90 | 0.62 | 0.43 | 0.78 |
| Dominica | DMA | B.1 | 1979 | 0.68 | 0.62 | 0.43 | 0.78 |
| Ecuador | ECU | A.1 | 2005 | 0.69 | 0.67 | 0.58 | 0.76 |
| Venezuela (Bolivarian Republic of) | VEN | B.1 | 1968 | 0.94 | 0.67 | 0.45 | 0.84 |
| Guam | GUM | B.1 | 1956 | 0.93 | 0.68 | 0.45 | 0.86 |
| Central African Republic | CAF | A.2 | 2012 | 0.40 | 0.69 | 0.56 | 0.8 |
| Benin | BEN | A.2 | 2013 | 0.47 | 0.71 | 0.64 | 0.77 |
| Democratic Republic of the Congo | COD | A.2 | 2012 | 0.36 | 0.71 | 0.64 | 0.76 |
| Colombia | COL | B.1 | 1995 | 0.76 | 0.72 | 0.6 | 0.82 |
| Gabon | GAB | B.1 | 1988 | 0.87 | 0.72 | 0.48 | 0.89 |
| Cameroon | CMR | A.2 | 2015 | 0.54 | 0.73 | 0.63 | 0.83 |
| Timor-Leste | TLS | A.2 | 2010 | 0.29 | 0.73 | 0.66 | 0.8 |
| Cook Islands | COK | B.1 | 1991 | 0.75 | 0.74 | 0.57 | 0.86 |
| Cote d'Ivoire | CIV | A.2 | 2015 | 0.53 | 0.74 | 0.61 | 0.85 |
| Martinique | MTQ | B.1 | 1990 | 0.89 | 0.78 | 0.62 | 0.89 |
| Solomon Islands | SLB | A.2 | 2015 | 0.22 | 0.78 | 0.69 | 0.86 |
| Ghana | GHA | A.2 | 2015 | 0.54 | 0.8 | 0.7 | 0.88 |
| Indonesia | IDN | A.2 | 2015 | 0.53 | 0.8 | 0.73 | 0.86 |
| Vanuatu | VUT | A.2 | 2015 | 0.26 | 0.8 | 0.72 | 0.86 |
| Philippines | PHL | A.2 | 2015 | 0.50 | 0.83 | 0.78 | 0.87 |
| Puerto Rico | PRI | B.1 | 1945 | 0.99 | 0.83 | 0.59 | 0.96 |
| Guadeloupe | GLP | B.1 | 1953 | 0.99 | 0.87 | 0.65 | 0.97 |
| Congo | COG | A.2 | 2012 | 0.65 | 0.89 | 0.83 | 0.93 |
| Singapore | SGP | B.1 | 1958 | 1.00 | 0.96 | 0.84 | 1 |
